# Exploring “big picture” scenarios for resilience in social–ecological systems: transdisciplinary cross-impact balances modeling in the Red River Basin

**DOI:** 10.1007/s11625-023-01308-1

**Published:** 2023-04-01

**Authors:** Anita Lazurko, Vanessa Schweizer, Derek Armitage

**Affiliations:** 1 School of Environment, Resources, and Sustainability, University of Waterloo, 200 University Avenue West, Waterloo, N2L 3G1 Canada; 2 Department of Knowledge Integration, University of Waterloo, Waterloo, Canada

**Keywords:** Social–ecological systems, Scenarios, Water governance, Resilience, Transdisciplinarity

## Abstract

Climate change is increasing the frequency and the severity of extreme events in river basins around the world. Efforts to build resilience to these impacts are complicated by the social–ecological interactions, cross-scale feedbacks, and diverse actor interests that influence the dynamics of change in social–ecological systems (SESs). In this study, we aimed to explore big-picture scenarios of a river basin under climate change by characterizing future change as emergent from interactions between diverse efforts to build resilience and a complex, cross-scale SES. To do so, we facilitated a transdisciplinary scenario modeling process structured by the cross-impact balances (CIB) method, a semi-quantitative method that applies systems theory to generate internally consistent narrative scenarios from a network of interacting drivers of change. Thus, we also aimed to explore the potential for the CIB method to surface diverse perspectives and drivers of change in SESs. We situated this process in the Red River Basin, a transboundary basin shared by the United States and Canada where significant natural climatic variability is worsened by climate change. The process generated 15 interacting drivers ranging from agricultural markets to ecological integrity, generating eight consistent scenarios that are robust to model uncertainty. The scenario analysis and the debrief workshop reveal important insights, including the transformative changes required to achieve desirable outcomes and the cornerstone role of Indigenous water rights. In sum, our analysis surfaced significant complexities surrounding efforts to build resilience and affirmed the potential for the CIB method to generate unique insights about the trajectory of SESs.

**Supplementary Information:**

The online version contains supplementary material available at 10.1007/s11625-023-01308-1.

## Introduction

Global climate change is expected to increase the frequency and severity of extreme events and shift the climatic regime in river basins around the world (IPCC 2021). This hydroclimatic intensification is significant and deeply uncertain (Milly et al. 2008; Marchau et al. 2019), exacerbating the risk of flood and drought damages, disruptions to food production and ecosystem services, and harms to human health (Rockström et al. 2014; Ray et al. 2019; IPCC 2021). The capacity of society to prepare for and cope with these risks depends upon several uncertain social and economic factors (Gallopín 2006; Engle and Lemos 2010), which are both made vulnerable by climate change and may further degrade natural river basin functions, such as through land use change (Lambin and Meyfroidt 2010). Resilient water systems also play a critical role in society’s capacity to deal with stresses and shocks more broadly (Falkenmark et al. 2019).

A shift in water governance is required to address the novel uncertainties and complexities introduced by climate change at a river basin scale. Water governance is defined broadly as the social functions that regulate and coordinate water development (Jiménez et al. 2020). The dominant nineteenth- and twentieth-century paradigm of water governance enabled rapid economic development but was limited by silo thinking, reactive management of externalities, and rigid control of variability (Pahl-Wostl 2007; Baird and Plummer 2021). For example, large-scale channels and dams enabled agricultural and energy production but were optimized for historical climate variability and may be brittle to climate change (Altinbilek 2002; McCartney 2009; Giuliani et al. 2016). In recent decades, various paradigms surfaced to deal with these challenges, such as Integrated Water Resources Management (Biswas 2008), the water–energy–food nexus (Benson et al. 2015), and adaptive governance (Folke et al. 2005; Huitema et al. 2009).

Most recently, the resilience paradigm (Walker et al. 2004; Folke et al. 2010) has been applied to enable effective water governance under climate change (Baird and Plummer 2021). From this view, river basins are complex social–ecological systems (SESs) that evolve with and adapt to environmental change, and outcomes emerge from social–ecological interactions and feedbacks across scales (Rockström et al. 2014; Walker 2020; Chester et al. 2021). Resilience here is “the capacity to adapt or transform in the face of change… particularly unexpected change, in ways that continue to support human wellbeing” (Folke et al. 2016, p. 41). For example, water managers may develop adaptive rather than static plans, optimize infrastructure for multiple climate scenarios rather than one, or use ecosystems for their natural capacity to buffer variability alongside traditional infrastructure (Pahl-Wostl and Knieper 2014; Faivre et al. 2017; Marchau et al. 2019).

While a resilience paradigm may in theory be effective for dealing with climate change, efforts to build resilience in practice are complex and contested. Novel approaches may be viewed as risky (Jeffrey and Gearey 2006) and must contend with the institutional inertia of conventional approaches (Sendzimir et al. 2010; Mendez et al. 2012; Marshall and Alexandra 2016). For example, infrastructure financing mechanisms may be biased away from valuing the long-term, systemic impacts of resilient solutions (Lazurko and Pinter 2022). Additionally, despite a shared language of resilience, efforts to build resilience hold hidden tensions and trade-offs rooted in divergent perspectives and interests in the future (Leach 2008; Helfgott 2018). For example, questions of resilience to what and for whom surface assumptions about what constitutes a desirable resilient future, and the degree of transformation required to achieve it. Most actors lack the tools and the frameworks to anticipate and navigate the future in a manner that reconciles such diverse framings, scales, and drivers of change (Bai et al. 2016; Verburg et al. 2016). These challenges are particularly pronounced in contexts where building resilience may require transformative changes that shift pathways toward a profoundly new system (Folke et al. 2016; Pereira et al. 2021).

Scenarios are promising tools for explicitly engaging with complex and uncertain futures (Peterson et al. 2003; Miller et al. 2014; Bai et al. 2016). Scenarios are coherent, internally consistent, and plausible descriptions of the potential future trajectories of a system (Heugens and van Oosterhout 2001). Explorative scenarios (i.e., what could happen) have been used in river basin contexts to project how climatic and socio-economic change may impact water supply and demand, and normative scenarios (i.e., what we want to happen) are often used to develop investment strategies (Varis et al. 2004; Dong et al. 2013; Elsawah et al. 2020). Emerging studies combine explorative and normative scenarios through participatory methods to collaboratively envision and strategize pathways toward sustainable or resilient river basin systems amid top-down pressures (Schneider and Rist 2014; Carpenter et al. 2015; Hirpa et al. 2018), and a handful of studies focus explicitly on scenarios related to resilience (e.g., Helfgott 2018).

Semi-quantitative scenario methods like the cross-impact balances (CIB) method are uniquely positioned to model integrative and holistic scenarios that “get the big picture roughly right” (Polasky et al. 2020). CIB applies systems theory to generate internally consistent narrative scenarios from a network of interacting drivers of change (Weimer-Jehle 2006; Weimer-Jehle et al. 2016). CIB made its debut in the field of technological forecasting and has been applied in over 100 studies in dozens of fields including energy transitions and climate change research (Schweizer 2020; Weimer-Jehle et al. 2020; Weimer-Jehle 2023). Applications are also expanding toward a wider range of policy processes (Stankov et al. 2021; Kosow et al. 2022), including in water management (e.g., Schütze et al. 2019; Motschmann et al. 2022). However, this relatively new method has evolved within its own community of practice and has yet to become established in SES research, despite its compatibility with SES theory. CIB is compatible with an SES perspective because it takes complexity seriously, modeling scenarios as emergent outcomes of systemic interactions and feedbacks (Kosow and Gaßner 2008), including across scales (Schweizer and Kurniawan 2016; Kemp-Benedict et al. 2019). This lies in contrast to the more popular Intuitive Logics (IL) method that develops four narrative scenarios by exploring the systemic consequences of the intersection of two drivers of change (Ramirez and Wilkinson 2014).

Additionally, while popular Story-and-Simulation approaches translate qualitative scenarios into inputs for quantitative models (Alcamo 2008; Elsawah et al. 2020), CIB integrates qualitative alongside quantitative drivers within the scenario model. Thus, CIB reconciles trade-offs between qualitative and quantitative methods that make big-picture scenario modeling challenging; quantitative methods may be data-informed and reproducible but exclude drivers of change or perspectives that are not measured in quantitative terms (Gerst et al. 2014; Moallemi et al. 2021), and qualitative scenario methods consider a wider range of future conditions, but, at times, lack the systematic analysis and analytical insights (e.g., model sensitivity analysis) promoted by quantitative methods (Ramirez and Wilkinson 2014).

Transdisciplinary scenario processes offer further opportunities to explore diverse perspectives and interests in the future of river basins. Transdisciplinary research caters to the problem-oriented and integrative nature of sustainability science by bringing together diverse actors to generate knowledge (Lang et al. 2012; Brandt et al. 2013). Participatory scenario models can be used to structure transdisciplinary research processes, in which a model is co-produced through engagement with collaborators (McBride et al. 2017; Voinov et al. 2018; Moallemi et al. 2021). The goal of such processes is not only to structure models and produce outputs but to mobilize knowledge in a way that facilitates societal impact and promotes meaningful learning for both scientists and participants. The CIB method is often used to develop authoritative models through expert-driven scenario development processes. However, emerging applications of CIB aim to facilitate stakeholder engagement and collaborative learning, opening up the method to a wider range of non-expert participants (e.g., Stankov et al. 2021; Sun 2021).

To our knowledge, few or no studies have applied the CIB method to model river basin scenarios under climate change, no studies have used CIB to explicitly model scenarios as emergent from complex social–ecological dynamics, and while CIB has been used in a participatory manner, it has not been used to explicitly structure transdisciplinary research. In this study, we aimed to explore big-picture scenarios of a river basin under climate change by characterizing future change as emergent from interactions between diverse efforts to build resilience and a complex, cross-scale SES. We also aimed to explore the potential for the CIB method to surface diverse perspectives and drivers of change in SESs through a transdisciplinary scenario modeling process.

## Methods

The principles of an ideal–typical transdisciplinary research process guided the study (Lang et al. 2012), including (a) case study formulation and collaborative problem framing, (b) efforts to co-create knowledge where feasible, and (c) seeking opportunities to (re)integrate the knowledge.

### Case study formation and collaborative problem framing

The participatory scenario modeling process was situated in the Red River Basin (RRB). The RRB is part of the Hudson Bay drainage system, covering parts of Minnesota, South Dakota, and North Dakota, before meandering northward for approximately 480 km into Lake Winnipeg in Manitoba (Red River Basin Commission 2005; Leitch and Krenz 2013). The RRB is governed by a complex arrangement of institutions from community to federal and transboundary level (Hearne 2007), and is the homeland of diverse First Nations, Métis, and Tribal communities including Cree, Ojibway, Anishinaabee, and Dakota communities. Climate change is expected to exacerbate existing climatic variability and its implications (Prairie Climate Centre 2013; Rasmussen 2016; Bertrand and Mcpherson 2018; Shrestha et al. 2020). Additional pressing issues include the eutrophication of downstream water bodies (Schindler et al. 2012) and soil erosion (Liu et al. 2015). A history of forced relocation and colonization of Indigenous lands in addition to contemporary socio-economic trends, such as agricultural technology and urbanization, introduce significant complexity to decision-making. Additionally, actors are attempting to build a more resilient system, such as by rehabilitating ecosystems and shifting toward regenerative agriculture, as discussed at the Annual RRB Land and Water Conference in January 2021.

The Red River Basin Commission (RRBC) and the International Institute for Sustainable Development were chosen as collaborators due to their active role in networks driving resilience-building efforts. Through consultation with these partners, the transdisciplinary scenario process was framed around the issue of resilience to ongoing floods and droughts. The year 2050 was chosen as the single temporal scale. This was chosen to situate the scenarios far enough in the future to ensure divergence from the present and to focus on linking across spatial scales, i.e., by simplifying the temporal scales (Scholes et al. 2013). A case study advisory committee of four individuals from various institutions in the basin was consulted throughout the research process.

### Co-creating knowledge through participatory scenario modeling

#### Cross-impact balances scenario method

The cross-impact balances (CIB) method projects internally consistent scenarios from a network of interacting drivers of change or critical uncertainties (Weimer-Jehle 2006; Kosow and Gaßner 2008). A CIB modeling process begins with determining a set of *descriptors*, which are the most important and uncertain drivers of change influencing the future of a system. The uncertainty of each descriptor is represented by a small number (i.e., 1–4) of *variants*, or mutually exclusive outcomes. In CIB, a scenario is made up of the selection of one variant for each descriptor. The systemic interactions between descriptors are determined by considering *influence judgments* between variants. These judgments are the direct influences of the selection of a variant from one descriptor on the selection of a variant from another. Influence judgments are captured in a judgment section, as depicted in Table 1, in which variants in the row are promoting (+) or inhibiting (−) variants in the column, on a scale of weak (1), moderate (2), or strong (3). Interactions with no direct influence are given an influence judgment of zero. According to best practice, each row in a judgment section should sum to zero (i.e., as depicted in Table 1) to satisfy the principle that the direct influence of the variant in the row is a source of selectivity between mutually exclusive variants in the column. The influence judgments for the whole system are captured in a *cross-impact matrix*.

**Table 1:** Example of a judgment section in a CIB matrix

	Descriptor 1		
Descriptor 2	Variant a	Variant b	Variant c
Variant *x*	+ 1	+ 2	− 3
Variant *y*	− 2	− 1	+ 3
Variant *z*	− 1	0	+ 1

Internally consistent scenarios are the stable or self-reinforcing configurations of the model, in which each descriptor exists in one of its variants. A software like ScenarioWizard (Weimer-Jehle 2021) is used to calculate the impact balances for each possible scenario to determine which scenarios are internally consistent (i.e., self-reinforcing and stable) or internally inconsistent (i.e., transient or unstable). Scenarios that are internally consistent are considered plausible by many CIB analysts (Schmidt-Scheele 2020). A full description of the mathematics of impact balances and internally consistent scenarios can be found in Weimer-Jehle (2006).

#### Social–ecological scenario framework

**Fig. 1:**
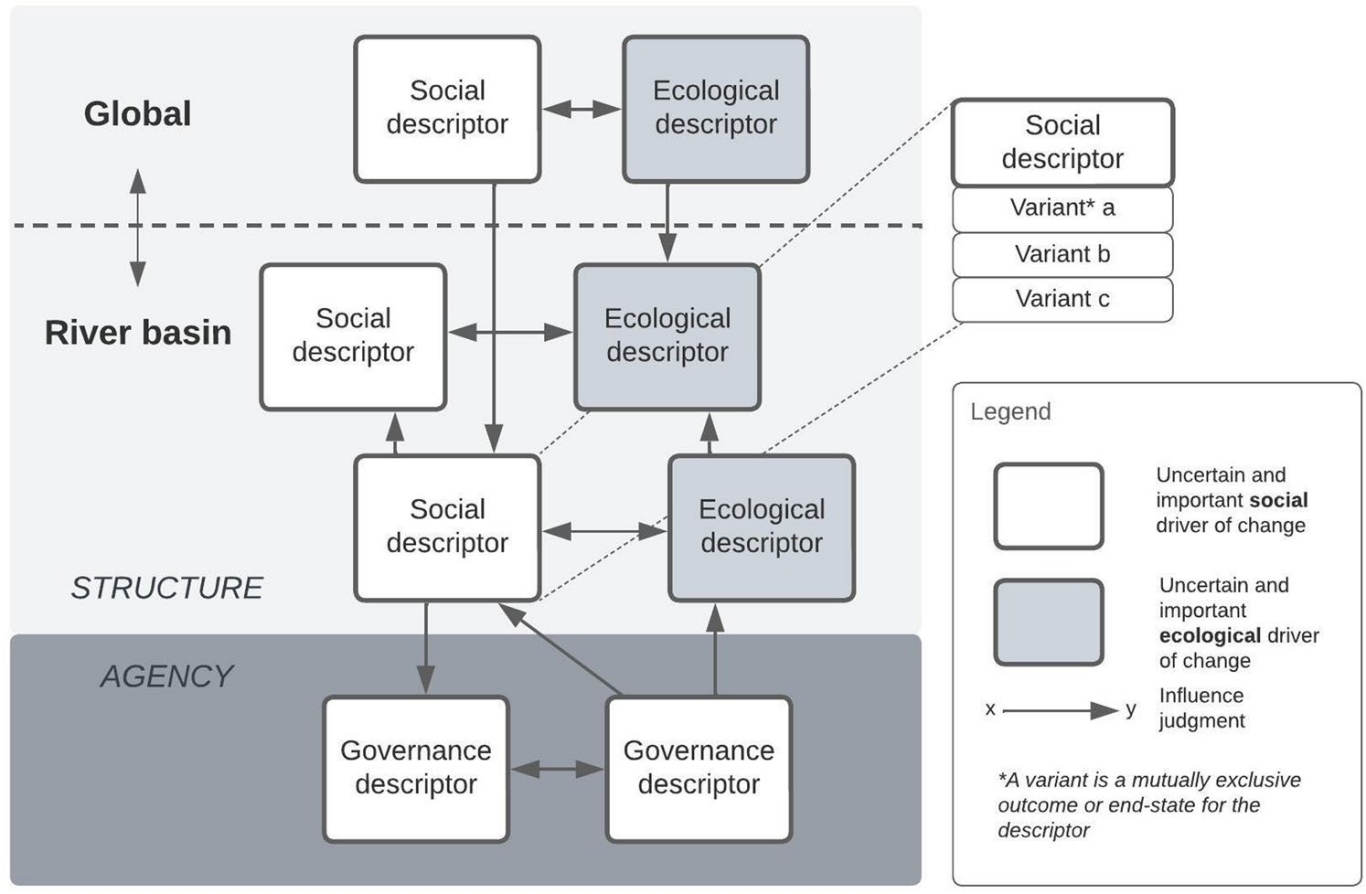
Social–ecological scenario framework

A guiding framework for the scenario modeling process was developed to characterize future change as emergent from efforts to build resilience and a complex, cross-scale SES. We call this framework the ‘social–ecological scenario framework’, depicted in Fig. 1. The framework brings together existing knowledge about the dynamics of SES change and the structure of the CIB method to allow for systematic and transparent scenario development.

The framework depicts the future of a SES as emerging from social–ecological interactions across scales (i.e., river basin and global) and between the system structure and actor agency. Cross-scale dynamics are depicted as the influence of global change on the river basin scale (Walker et al. 2006; Scholes et al. 2013; Reyers et al. 2018). More specifically, social and ecological subsystems (i.e., social and ecological descriptors, variants, and their interactions at the river basin scale) are influenced by broader social, economic, and political settings and related ecosystems (i.e., social and ecological descriptors, variants, and their interactions at the global scale), as per the seminal framework for analyzing the sustainability of SES (Ostrom 2009; McGinnis and Ostrom 2014).

While the CIB method evaluates the plausibility of scenarios (i.e., as internal consistency), balancing scenario plausibility with diversity is important to capture the potential for social–ecological transformation. According to SES theory, transformative change emerges from the interplay of top-down structural change with bottom-up actor agency (Moore and Westley 2011; Westley et al. 2013). Thus, actor agency is represented in governance descriptors, which interact with structural social–ecological descriptors. In addition, seeds (i.e., small-scale yet promising innovations in the present assumed to be mainstream in future) were introduced as variants to the governance descriptors to reflect the view that transformation emerges when these marginal innovations interact with top-down structures to scale in higher level systems (Geels 2002; Moore et al. 2014; Bennett et al. 2016).

A key characteristic of complex SESs is emergent outcomes (Reyers et al. 2018; Schlüter et al. 2019). In the framework, interactions between efforts to build resilience and the social–ecological context implicate social–ecological, cross-scale, and structure–agency interactions, producing emergent internally consistent scenarios. Thus, the cross-impact matrix that details these interactions defines a stability landscape for the SES, and the internally consistent scenarios are the stability domains or basins of attraction (Walker et al. 2004; Folke et al. 2010). Changes to the influence judgments in the cross-impact matrix alter the stability domain, shifting the internal consistency of scenarios and thus generating new or altered basins of attraction.

#### Participatory scenario modeling

**Fig. 2:**
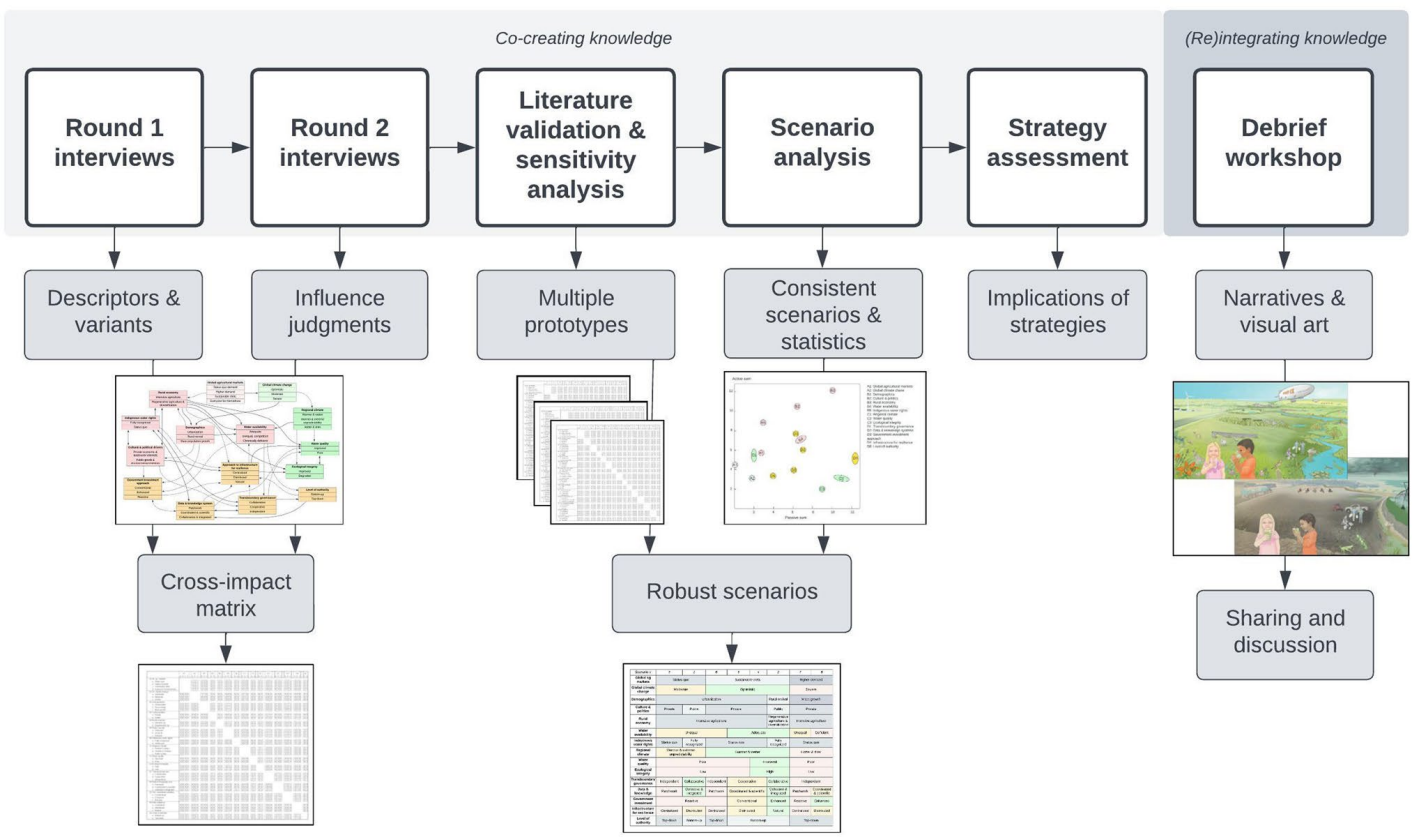
Six-step transdisciplinary scenario modeling process

A summary of the six-step participatory scenario modeling process is depicted in Fig. 2.

##### Round 1 interviews 

Semi-structured interviews with 34 experts and opinion leaders in the RRB elicited critical social and ecological drivers of change influencing resilience to climate variability and change, visions of a desirable resilient future, and current practices or projects contributing to that future. Prior to the interviews, a scan of academic and gray literature generated a preliminary list of descriptors. The documents were gathered from case study partners and relevant keyword searches on Web of Science, Scopus, and Google Scholar, and broad themes were translated into descriptors for each category in the social–ecological scenario framework.

Consultation with study partners generated an initial list of interviewees. Snowball sampling determined additional interviewees until reaching an approximate saturation point. The 34 interviewees were recruited to represent various levels of governance and areas of expertise, with some interviewees representing multiple perspectives. The levels of governance represented include transboundary (10), federal (8), provincial or state (11), municipal or watershed (7), Indigenous organization or governance (5), and general experts (9). Interviewees were experts or opinion leaders (i.e., a mixture of academics and practitioners) on at least one of the following: agriculture (8), climate (11), environment and ecology (15), governance (12), water management and infrastructure (17), and Indigenous governance (6). Indigenous perspectives were included through academic experts on Indigenous governance rather than Elders due to concerns regarding the ethics of coding Indigenous knowledge. Efforts were made to get participants from both the US and Canada, but a majority (22) of the interviewees were Canadian. Participation challenges were exacerbated by the availability of interviewees, particularly as the study was conducted during the COVID-19 pandemic.

Interviews were conducted virtually with a Miro board following the interview protocol in S1. The preliminary descriptors from the document scan were used as examples to prompt discussion, but participants were encouraged to generate additional descriptors. The interviews were audio recorded and transcripts were coded in NVivo in three rounds. In the first round, interviewee responses were coded into general themes that roughly fit as descriptors in the main categories of the social–ecological scenario framework. Interviewee responses to questions regarding current practices or projects contributing to resilience were coded as potential seeds. In the second round, the text under each category of the model framework was coded with the structure of CIB in mind to generate a provisional set of descriptors and variants, introducing seeds as variants where appropriate. Attempts to draft a description for each descriptor and variant alongside a third round of coding refined the list to 15 descriptors, each with 1 to 4 variants. While effective, the process of coding interviews inevitably introduced subjectivity due to the interpretation required to translate interviewee knowledge into the structure of a CIB model. These descriptors and variants were discussed with the case study advisory committee and circulated to all participants for feedback, resulting in some minor adjustments.

##### Round 2 interviews

A second round of semi-structured interviews with 11 experts and opinion leaders elicited the influence judgments to complete the cross-impact matrix (i.e., the stability landscape of the SES). Before the interviews, the transcripts from round 1 interviews were coded a fourth time to identify relationships between descriptors. Interviewee statements that clearly indicated the direction and approximate strength of the interaction were translated into provisional influence judgments and depicted in a network diagram. Uncertain judgment sections were highlighted to prioritize the discussions during round 2 interviews.

Round 2 interviewees were topical experts selected in consultation with partners. Ten of these interviewees had participated in round 1 interviews and all were selected due to specific expertise in at least two descriptors in the model associated with uncertain influence judgments. These interviews continued until all uncertain judgment sections were discussed. Again, interviews were conducted virtually with a Miro board following the interview protocol in S2. The interviews were targeted to the most uncertain influences in the model and the expertise of the interviewee. Interviewees who found the language and structure of CIB intuitive were asked directly for influence judgments, while others were asked to describe interactions qualitatively. These descriptions were later translated into influence judgments. At this stage, several judgment sections were still uncertain due to a wide range of ontological (i.e., inherent system variability) and epistemic uncertainties (i.e., lack of knowledge), in addition to ambiguity (i.e., divergent framings) (Dewulf and Biesbroek 2018).

##### Literature validation and sensitivity analysis

A review of targeted literature triangulated interview data and addressed remaining uncertain judgment sections. Because the range of topics addressed in the CIB model was broad, literature was generated by (a) revisiting documents from the document scan informing round 1 interviews and (b) searching Google Scholar, Scopus, and Web of Science for keywords relevant to the topics for each judgment section. A scan of the title and abstract of the results determined which results were read in detail to find supporting evidence for various influence judgments. Literature from the RRB context was used where possible, but other regional or global literature was used when appropriate.

Because most interviewees were experts on the river basin scale, literature on the shared socio-economic pathways (SSPs) was the sole data source for characterizing influence judgments between descriptors at the global scale. The SSPs depict plausible socio-economic futures at the global scale for use in climate change research (Nakicenovic et al. 2014; van Vuuren et al. 2017). Several studies characterize the implications of different SSPs on the regional scale (e.g., high-, middle-, and low-income countries) in a manner that links global descriptors, such as agricultural markets, to river basin scale descriptors, such as the state of the rural economy (e.g., Calvin et al. 2017; Graham et al. 2018). The SSPs also indicate how different socio-economic descriptors like global agricultural markets may contribute to greenhouse gas emissions. Thus, the SSPs were also used to link global descriptors with global climate change. The rationale for these judgment sections is detailed alongside all other judgment sections in S3.

The sensitivity analysis of the remaining uncertain influence judgments followed the protocol described by Schweizer and Kriegler (2012). The style of sensitivity analysis is atypical, because it was not used solely to assess the sensitivity of the model to changes in input data. Rather, we focused on how input data for CIB also specifies the structure of a network of system influences (i.e., as determined by the influence judgments in the CIB matrix), which means that changes to input data for CIB also changes the model structure. Our sensitivity analysis focused on how such changes in model structure (i.e., due to different influence judgments) generate new output data (i.e., internally consistent scenarios), thus allowing us to identify scenarios that are ‘robust’ to model uncertainty (i.e., to find scenarios that emerge as internally consistent regardless of the ‘sensitivities’ in the model data). The protocol begins with designating a baseline model void of uncertain influence judgments and then identifies each uncertain influence judgment as a type I (i.e., new influences from baseline), type II (i.e., adjusted relationships from baseline), or type III (i.e., selected combinations of influences) sensitivity analysis. The numerous sensitivities these three types explored were configured into six independent prototypes of the model that represented the maximally diverse range of uncertainty in the model. S3 describes the type of sensitivity for each uncertain judgment section, and S4 elaborates the sensitivity analysis protocol.

##### Scenario analysis

ScenarioWizard was used to generate internally consistent scenarios (i.e., basins of attraction of the SES) for each prototype. The scenario analysis focused on determining which internally consistent scenarios were common across model prototypes and are thus robust to model uncertainty (i.e., valid regardless of ‘sensitivities’ in the model data). The frequency statistics, bias statistics, and the active–passive diagrams for the different prototypes generated further insights. Bias statistics are used to check the quality of influence judgments by revealing systemic bias away from consistent scenarios containing a specific variant (Weimer-Jehle 2021). A bias statistic of less than 10% was considered an indication of significant bias in the model that should be re-examined for quality assurance. An active–passive diagram depicts the role of descriptors within the system. The active sum (*y*-axis) represents the degree to which the descriptor is an impact source (i.e., exerts influences on other descriptors). The passive sum (*x*-axis) represents the degree to which the descriptor is an impact sink (i.e., receives influences from other descriptors). The findings of these methodological steps for scenario analysis are discussed in “Results and discussion” section.

##### Strategy assessment

In addition to the scenario analysis, the implications of three water governance strategies were tested in the model. These strategies were discussed by many interviewees as potentially influential shifts in the system but were not characterized in the model structure. Assumptions were made about how select influence judgments may change, generating a new model prototype for each strategy (i.e., transformations in the stability landscape of the SES, generating new basins of attraction). These adjustments created new prototypes for each strategy, which were then modeled in ScenarioWizard. The results were compared to the original six prototypes, focusing on internally consistent scenarios, frequency statistics, and bias statistics.

### Knowledge (re)integration

The final phase of knowledge (re)integration first required translating model outputs into formats that could be shared to stimulate discussion among participants. Five of the most divergent scenarios were selected from the eight robust scenarios to offer a manageable number for participants to discuss. Outputs from ScenarioWizard include combinations of descriptor variants (i.e., scenarios) as well as the particular causal chains (i.e., the series of if–then logic statements encoded as influence judgments). These outputs are embedded in the cross-impact matrix and underlie why the combinations emerge as internally consistent scenarios. The lead author translated such outputs from ScenarioWizard into narratives. The narratives describe the RRB under each chosen combination of descriptor variants, highlighting key influences that contribute to the internal consistency of the scenario. A local artist depicted these scenarios as visual art.

#### Debrief workshop

A virtual workshop aimed to facilitate deeper engagement with the results. While the initial intention of the workshop was to ‘reintegrate’ knowledge as per the ideal–typical research process (Lang et al. 2012), time and format constraints led to a more traditional knowledge sharing workshop. Twenty-two participants were recruited from interviewees and the board of the RRBC. Nineteen of the 22 participants had participated in at least one round of interviews. As in the interviews, participants represented various levels of governance (with some representing multiple), including transboundary (9), federal (5), provincial or state (12), municipal or watershed (6), academic experts on Indigenous governance (4), and general experts (5). Workshop participants were experts or opinion leaders on at least one of the following: agriculture (3), climate change (7), environment and ecology (8), governance (7), water management and infrastructure (17), and Indigenous governance (5).

The workshop began with a presentation of the rationale, methodology, and results, using the narratives and visual art to communicate the scenarios. Participants were then split into breakout rooms where they worked together to (1) rank scenarios from most to least desirable, (2) rank scenarios from most to least plausible, and (3) discuss how existing initiatives are promoting or inhibiting different scenarios. In a final debrief, participants were asked whether the scenario process changed the way they thought about the future of the RRB. This question served as a simple evaluation in the absence of more robust pre- and post-workshop surveys. The full workshop protocol is included in S6.

The workshop transcripts were analyzed using a simple thematic content analysis. The analysis focused on statements that surfaced potentially divergent assumptions about scenario desirability and plausibility, in addition to statements linking existing initiatives to the scenarios. Participant responses to the debrief questions were analyzed to provide a broad indication of the extent to which the CIB method effectively helped actors explore diverse perspectives and drivers of change in a SES.

### Researcher positionality

Researcher positionality is important for transdisciplinary research in contexts with diverse perspectives and interests like the RRB. The lead author who conducted fieldwork and interpreted the data is a western-trained scientist and Canadian settler. While efforts were made to avoid scientific subjectivity and bias, this positionality may have influenced access to study participants, the information participants felt comfortable to share, and how different perspectives (e.g., scientific versus local or practitioner knowledge) were interpreted and integrated into the scenario model. These biases may have also been influenced by the virtual format of the study, which allowed for the use of novel tools (e.g., Miro boards during interviews and workshops) while limiting recruitment of participants to those who were comfortable with and available for online engagement during the COVID-19 pandemic.

## Results and discussion

This section summarizes the study results and the significance of the findings for the phases of co-creating knowledge (“Co-creating knowledge: participatory scenario modeling” section) and sharing and (re)integrating knowledge (“Knowledge (re)integration: debrief workshop” section).

### Co-creating knowledge: participatory scenario modeling

#### Descriptors and variants

The 34 round one interviews generated fifteen descriptors, which are the important and uncertain drivers of change relevant to resilience to climate variability and change in the RRB. Social and ecological drivers of change at river basin and global scales make up the structure of the cross-scale SES, and governance descriptors characterize efforts to build resilience, introducing the influence of actor agency. Multiple variants for each descriptor cover a range of mutually exclusive outcomes. Several seeds were included as variants (e.g., “collaborative governance” under the transboundary governance descriptor), broadening the scope of outcomes to include the potential for transformation. Detailed descriptions of these variants are summarized in Table 2.

**Table 2:** Descriptors and variants according to categories in the social–ecological scenario framework, including scale, structure (S) and agency (A), and type (social or ecological)

Scale	S/A	Type	Descriptor	Variants	Details
Global	Structure	Social	Global agricultural markets Direct influences of changing global agricultural market demand on the RRB	Status quo	Stable demand for conventional agricultural exports. (In a world in which social, economic, and technological trends do not shift significantly from historical patterns, demand for agricultural exports remains stable.)
				Increasing demand	Higher demand for conventional agricultural exports. (In a high-economic-growth future driven by status quo consumption, rapid development increases food demand globally.)
				Sustainable diets	Higher demand for agricultural exports that meet environmental standards. (In a sustainable future, average global food demand is high as poverty reduction continues, with a shift in consumer preference for sustainable and plant-based diets
				Everyone for themselves	Agricultural demand primarily within Canada and the US, resulting in lower demand for agricultural exports from the RRB. (In a future with resurgent nationalism and security concerns, countries pursue food and energy self-sufficiency, isolating markets to regional production, depressing global demand for exports.)
Global	Structure	Ecological	Global climate change Global greenhouse gas (GHG) emissions scenarios	Optimistic RCP^a^ 1.9–2.6	Net zero emissions by 2050, curbing severe climatic shifts. Moderate impacts of climate change by 2050
				Middle-of-the-road RCP 4.5	Emissions remain at 2015 levels to 2050. Significant impacts of climate change by 2050
				Severe RCP 7.0–8.5	Emissions continue to rise and double by 2050. Severe impacts of climate change by 2050
River basin	Structure	Social	Demographics Population growth and distribution in the RRB	Urbanization	Moderate population growth (within projected range^b^) in urban centers. Continued rural depopulation
				Rural revival	Moderate population growth (within projected range^b^) with outmigration to rural areas. Revival of rural economy and cultural life
				Mass population growth	Significant population growth (exceeds projected range^b^) driven by migration to both rural and urban areas
River basin	Structure	Social	Cultural and political drivers Dominant cultural and political priorities driving decision-making in the RRB	Private landowner and economic interests	Decision making prioritizes private landowner interests and economic growth. Environment only restored or protected if near-term business case
				Public goods and environmental interests	Decision making prioritizes public goods and environmental interests over private interests when required. Environment restored or protected to enable long-term resilience of the economy and ecosystems
River basin	Structure	Social	Rural economy Dominant economic sectors driving the rural economy in the RRB	Intensive agriculture	Economy of the RRB continues to be driven by intensive agriculture on increasingly large-scale farms. Technological developments like precision agriculture offer opportunities for economic efficiencies
				Regenerative agriculture and diversification	Economy of the RRB shifts toward regenerative agriculture and diversifies (either within the agricultural sector or beyond). Technological developments like precision agriculture offer opportunities for improved soil health and sustainability. Economy diversifies to include smaller-scale agriculture and local processing
River basin	Structure	Social	Water availability Reliability of water availability to meet demand in the RRB (quantity only)	Adequate	Water availability generally adequate for demand. Seasonal deficiencies or issues manageable
				Unequal	Water availability insufficient for demand in some seasons or locations. Deficiencies or issues unmanageable, leading to unequal access and competition across sectors and demographics
				Chronically deficient	Water availability insufficient for demand across the RRB for multiple years in a row. Deficiencies and issues unmanageable, depressing economic activity. Higher risk of poor health outcomes, especially in rural areas
River basin	Structure	Social	Indigenous water rights Degree of recognition of Indigenous water rights and values in governance and management of the RRB	Fully recognized	Indigenous water rights are fully recognized. (e.g., prioritizing Indigenous interests over private interests when required, protecting the environment for its inherent value, and including Indigenous knowledge in decision-making.)
				Status quo	Indigenous water rights are not fully recognized, perpetuating the status quo. (e.g., de-prioritizing Indigenous interests, not recognizing inherent value of the environment, excluding lack of recognition of inherent cultural and social value of the environment, and excluding Indigenous knowledge in decision-making.)
River basin	Structure	Ecological	Regional climate Temperature and precipitation in the RRB under a changing climate	Warmer and wetter	Gradual increase of average annual temperature. Climate variability within recent historical range with overall increase of annual precipitation
				Warmer and extremely unpredictable	Gradual increase of average annual temperature. Increased atmospheric moisture content increases risk of rain-based summer flood events. Climate variability more extreme and unpredictable, with both floods and droughts possible within the same year
				Hotter and drier	Rapid and extreme temperature increase and/or shifting storm tracks lead to severe, multi-year or multi-decadal droughts
River basin	Structure	Ecological	Water quality Quality of water in the RRB and downstream water bodies (Lake Winnipeg)	Improved	Nutrient loading and pollution into the RRB reduced, improving the quality of water within the RRB and Lake Winnipeg
				Poor	Nutrient loading and pollution into the RRB continue at a status quo or increased rate, perpetuating contamination of water within the RRB and of Lake Winnipeg
River basin	Structure	Ecological	Ecological integrity Broad indicator for the integrity of natural ecosystems in the RRB, including their stability, dynamics, and “naturalness”	Improved	Ecosystem structure and function are restored or protected. Natural resilience to shock (climatic and other) is high. Low vulnerability to invasive species and pests
				Degraded	Ecosystem structure and function are disrupted and degraded. Natural resilience to shock (climatic and other) is low. High vulnerability to invasive species and pests
River basin	Agency	Governance	Transboundary governance Nature of transboundary governance in the RRB	Collaborative	Collaboration driven by shared goals between all parties. Municipal, state, provincial, Indigenous, and federal governance entities meaningfully included. Transboundary organizations act as enablers of initiatives
				Cooperative	Cooperation driven by independent goals and interests. Governance entities discuss when required, supported by formal agreements. Transboundary organizations are convenors of inter-jurisdictional discussions regarding transboundary issues or joint initiatives
				Independent	No meaningful cooperation. Governance entities act independently and without consultation. Formal transboundary agreements frequently broken to pursue domestic interests. Transboundary organizations are mediators of inter-jurisdictional conflicts
River basin	Agency	Governance	Data and knowledge systems Type of data and modeling systems in the RRB	Patchwork	Data collection is patchy. Modeling and forecasting is uncoordinated and without linkages to clear decision points. Climatic forecasting capacity not significantly improved. No forum for integration across scientific, local or Indigenous data and knowledge
				Coordinated and scientific	Data collection is comprehensive and shared across jurisdictions. Modeling and forecasting is coordinated basin-wide, with significant improvements in climatic forecasting. Modeling and forecasting have clear linkages to some decision points. No forum for integration across scientific, local, or Indigenous data and knowledge
				Collective and integrated	Data collection is organized and shared across jurisdictions. Modeling and forecasting is done collectively, with clear linkages to most decision points. Climate forecasting is significantly improved. Basin-wide forum for integration across scientific, local, and Indigenous data and knowledge established and maintained
River basin	Agency	Governance	Government investment approach Type of resilience measures supported by government investment bodies (provincial, state, federal)	Conventional	Government investments focus on gray/hard infrastructure measures with large financial disbursements and clear returns
				Enhanced	Government investments are more flexible to include soft (e.g., data monitoring), natural (e.g., wetland restoration), and gray (e.g., reservoirs) infrastructure measures. Investments disbursed in smaller increments with more flexible returns
				Reactive	Government investments focus reactively on emergency support and insurance, due to insufficient spending on maintenance, preventative measures
River basin	Agency	Governance	Approach to infrastructure for resilience Dominant approach to water resilience	Centralized infrastructure	Hydroclimatic variability (i.e., floods, droughts, and seasonal precipitation) managed with large-scale infrastructure, including reservoirs, diversions, and intra/inter-basin transfers. Continued drainage from landscape, including uncontrolled tile drainage. Highly managed ecosystems with few or none of them in their natural state
				Distributed infrastructure	Hydroclimatic variability managed with distributed system of ponds and controlled tile drainage. Large-scale infrastructure used to supplement distribution system if required. Highly managed ecosystems with few or none of them in their natural state
				Natural ecosystems	Hydroclimatic variability buffered with natural ecosystems. Controlled drainage and ponds manage extremes beyond natural ecosystems’ capacity when required. Large-scale infrastructure only built as a last resort. Significant restoration of natural ecosystems
River basin	Agency	Governance	Level of authority Dominant level of governance authority driving water resilience measures	Bottom-up, watershed	Bottom-up initiatives are dominant drivers of resilience. Continued or increased devolution of authority to local scales
				Top-down, state/province	Top-down initiatives are dominant drivers of resilience. Authority maintained at state/province or federal level, restricting local-level authority

^a^Representative concentration pathways (RCP) are greenhouse gas emissions concentration scenarios used by the IPCC. The numbers represent radiative forcing (Watts per meters-squared)

^b^Population projections for the Red River Basin are not readily available. Manitoba projects the population will grow from a baseline of 1.35 million (2017) to 1.57–1.95 million by 2043 (https://www150.statcan.gc.ca/n1/en/pub/91-520-x/91-520-x2019001-eng.pdf?st=uNlp1A0v). North Dakota projects the population will grow from a baseline of 0.67 million (2010) to 0.92–1 million by 2040 (https://www.commerce.nd.gov/census/). Minnesota projects the population will grow from a baseline of 5.89 million (2019) to 6.46 million in 2050

#### Influence judgments and multiple prototypes

**Fig. 3:**
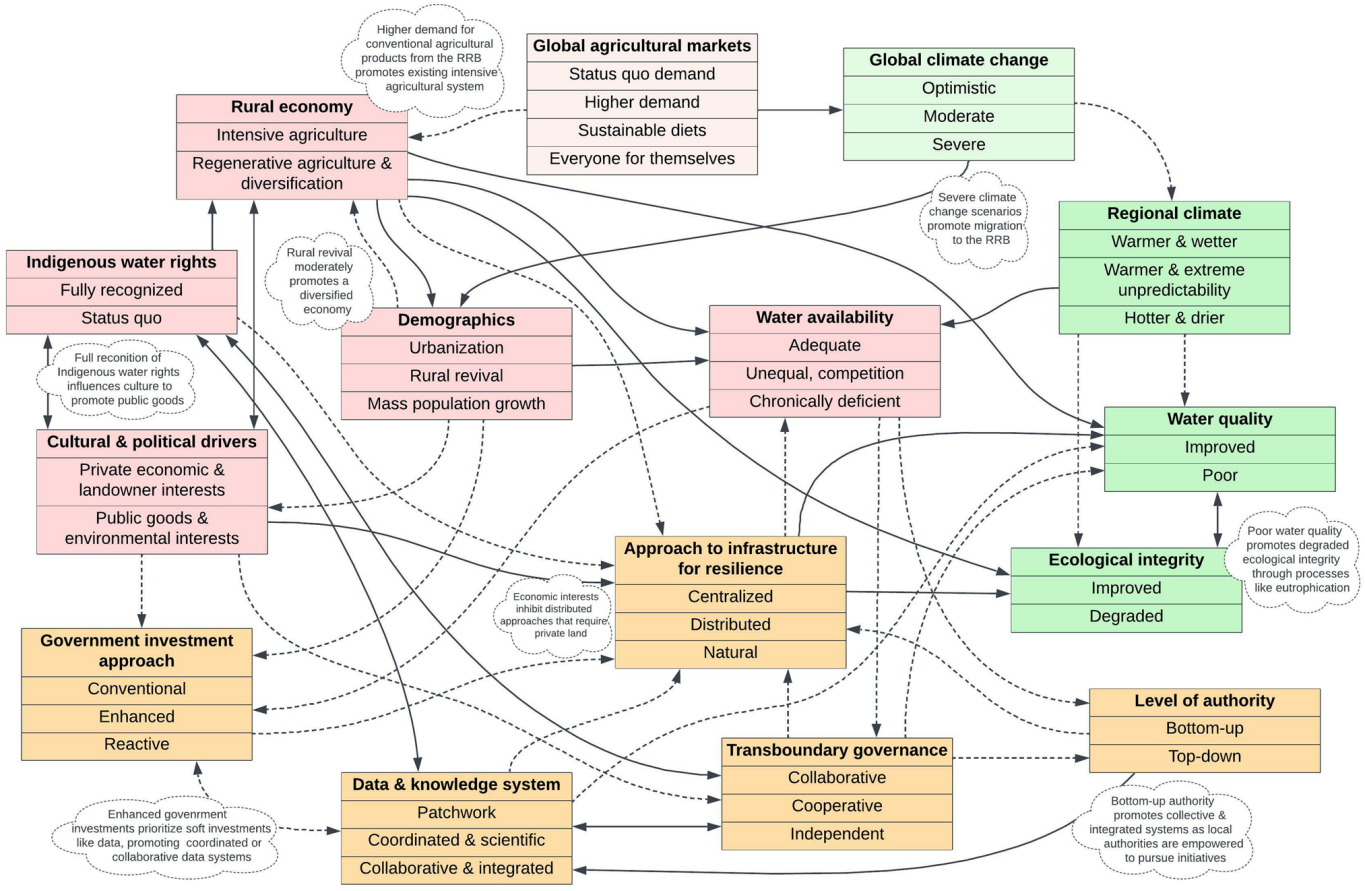
Descriptors, variants, and influence judgments in the scenario model. Arrows indicate judgment sections containing non-zero influence judgments. Dashed lines represent influence judgments that remained uncertain following the literature validation. Bi-directional arrows represent two influence judgments (i.e., in each direction), which are justified by different rationales

The influence judgments characterize social–ecological and cross-scale interactions between descriptors and variants, generating a stability landscape for the future of the RRB as depicted in Fig. 3. Arrows indicate judgment sections containing non-zero influence judgments between the connected descriptors. Following round 2 interviews, many influence judgments were uncertain, primarily due to a lack of clarity about the system (i.e., indistinctness, from interviewees and literature) and ambiguity (i.e., multiple interpretations of the system from interviewees and literature). For some influence judgments, such uncertainties persisted following the literature validation, which is represented with dashed lines. The influence judgments and their supporting evidence are summarized in S3. The sensitivity analysis generated six model prototypes, which together represent the maximally diverse range of uncertainty in the model.

#### Consistent scenarios and statistics

**Fig. 4:**
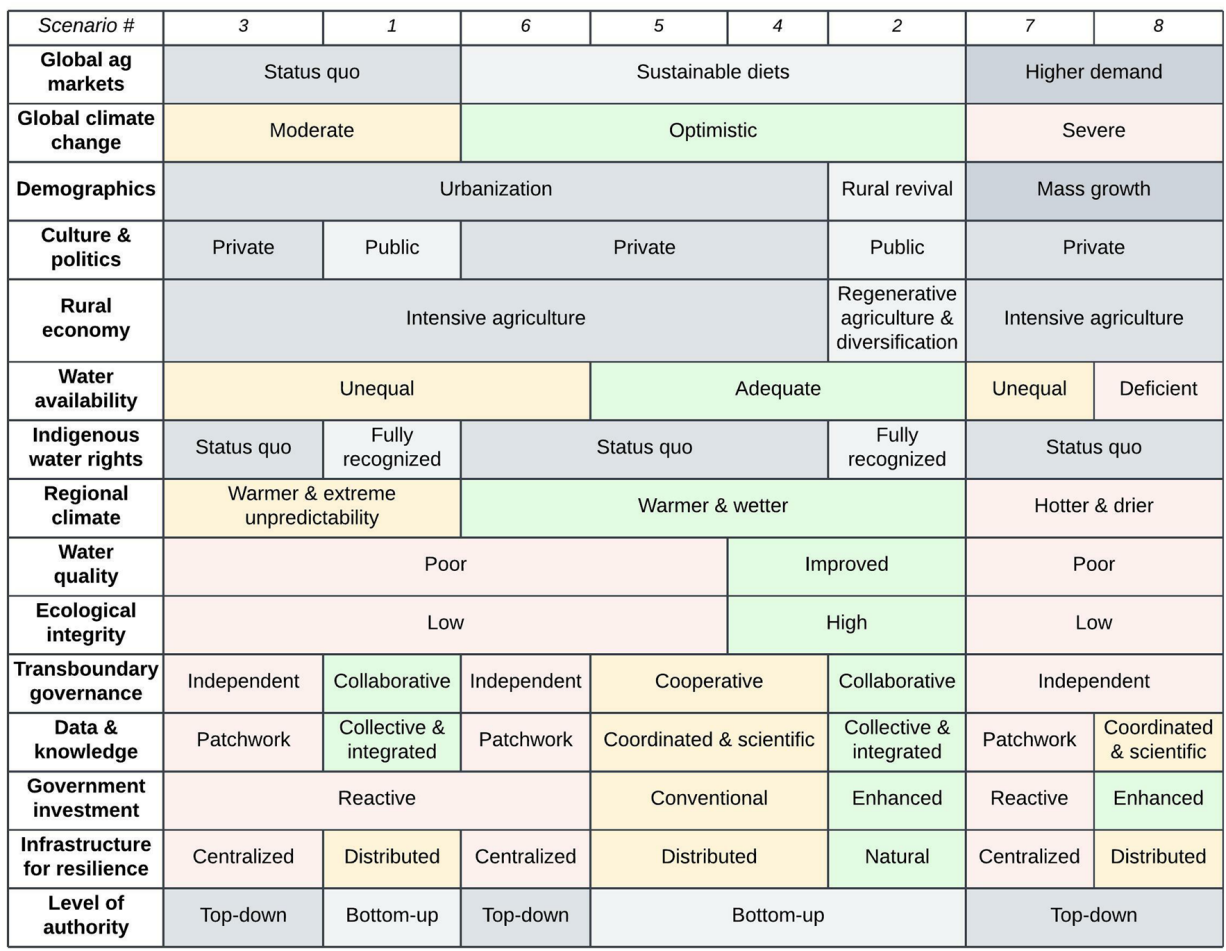
Scenario tableau depicting eight robust scenarios. Scenario numbers are listed along the top of the tableau (note: scenarios are not in numerical order as they were rearranged from their original ScenarioWizard output to improve readability)

The six prototypes were analyzed in ScenarioWizard, each generating 13–23 internally consistent scenarios. The scenarios describe big-picture futures for the RRB under climate change and are basins of attraction on the stability landscape of social–ecological interactions defined by the influence judgments in each prototype. Eight of these scenarios were robust to model uncertainty, depicted in the scenario tableau in Fig. 4. In the tableau, one scenario is described by the variants listed in a vertical column (enriched by the detailed descriptions for each variant in Table 2). A description of the robustness criterion, in addition to a broader set of seventeen scenarios that were less robust, are included in S5.

These scenarios reveal important insights into the future of the RRB under climate change. For example, independent governance, patchwork data, and other generally undesirable governance interventions tend to co-occur (e.g., scenario 3, 6, and 7), contributing to poor environmental outcomes. More desirable governance interventions, such as collaborative governance and integrated data, also co-occur (e.g., scenario 1, 2, 4, and 5), improving environmental outcomes. Still, the state of global descriptors has a strong influence over environmental outcomes, sometimes overshadowing positive governance interventions (e.g., scenario 1).

**Fig. 5:**
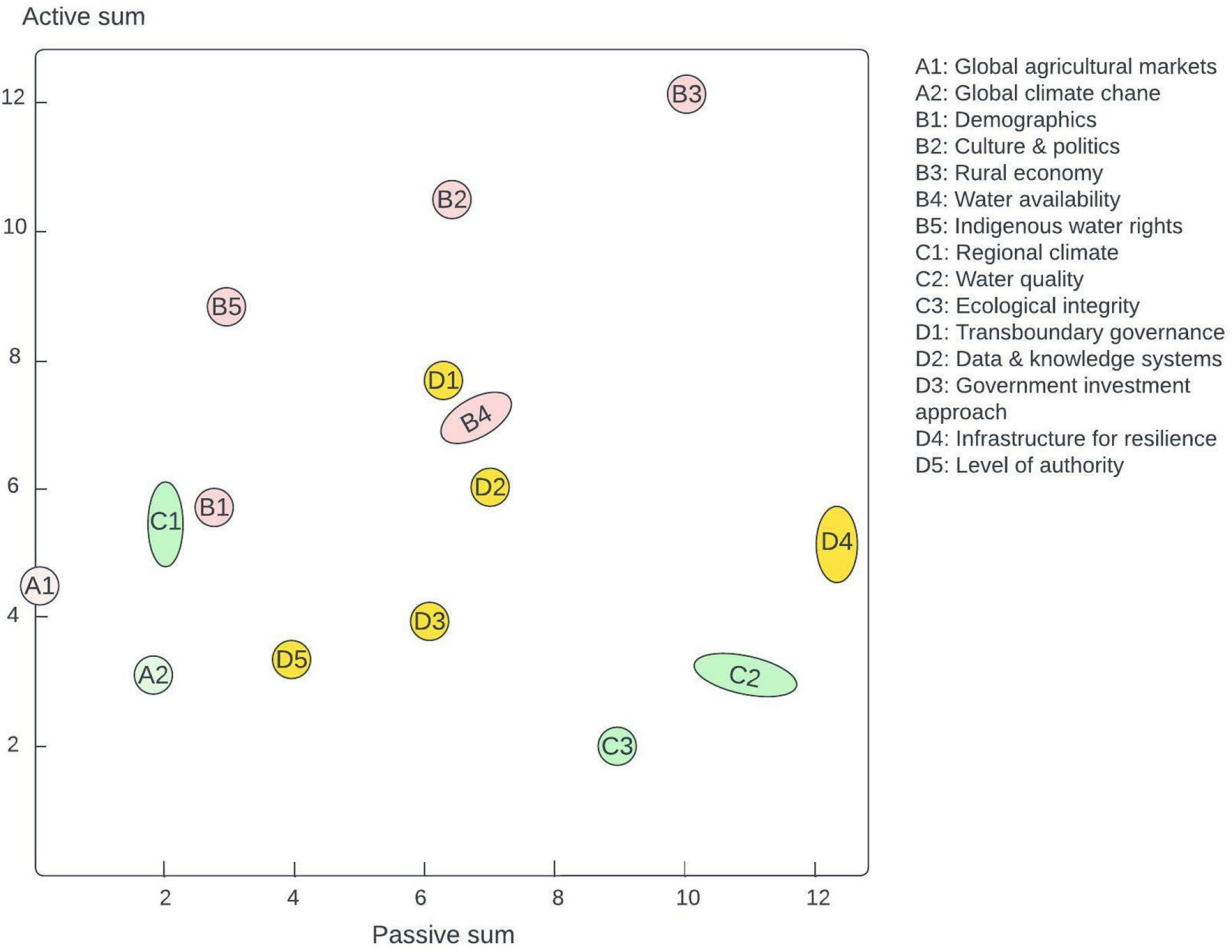
Active–passive diagram as discussed in the section "Consistent scenarios and statistics". Elongated circles represent deviations between prototypes

Figure 5 depicts the approximate active–passive diagram. Descriptors in the top left quadrant, such as Indigenous water rights, have a highly influential role on the system. Descriptors in the bottom right quadrant, such as water quality or ecological integrity, are strongly influenced by other descriptors in the system. Descriptors in the top right quadrant, such as the rural economy, are both strongly influencing and influenced by other descriptors, and thus are connected to complex system behavior (Weimer-Jehle 2021).

The only variant with a bias statistic of less than 10% is the ‘natural ecosystems’ approach to infrastructure for resilience (5.6–8.3%). This bias is due to restricting influences from several variants including an intensive agricultural economy, reactive or conventional government investment approaches, patchwork data and knowledge systems, and private economic and landowner interests. Correcting this bias by adjusting the influence judgments associated with this variant would require changes that deviate significantly from supporting evidence, so the bias was accepted as possibly representing true causal relationships for the analysis.

Together, the bias statistics and active–passive diagram reveal important implications for resilience in the RRB. The presence of strong restricting influences on the natural ecosystems approach to infrastructure for resilience indicates that transformative change may be required in several areas for a natural ecosystems approach to be mainstream. Additionally, the complex behavior associated with the role of the rural economy (i.e., due to its combined active *and* passive role in the system) reveals that isolated efforts to shift away from an intensive agricultural economy may have unexpected consequences. Importantly, the active role of Indigenous water rights, in combination with the concurrence of full recognition of Indigenous water rights with more desirable governance outcomes, reveals its potentially cornerstone role in realizing desirable outcomes overall.

#### Implications of strategies

The strategy assessment evaluated a collaborative response to scarcity, true market for ecological goods and services, and effective demand management, as described in Table 3. These strategies redefined specific influence judgments, shifting the stability landscape of social–ecological interactions in ways that may contribute to transformative change. S7 details the rationale for the changes in influence judgments.

**Table 3:** Summary of redefined influence judgments in strategy assessment

Strategy	Description
Collaborative response to scarcity	Chronically deficient water availability promotes (instead of restricts) collaborative and cooperative transboundary governance
True market for ecological goods and services	Ecosystem goods and services are valued in the economy (e.g., water quality and ecosystem services markets); private economic interests promote (instead of restricting) regenerative agriculture and distributed/natural approach to infrastructure
Effective demand management	State of the rural economy and demographics no longer directly influence water availability

Many participants discussed the importance of a *collaborative response to scarcity*, but the model showed that collaboration alone does not change outcomes in all cases. For example, under the assumption that the centralized infrastructure approach is the most effective for resilience, some chronically deficient water availability outcomes are avoided, but governance outcomes deteriorate. This surprising result is due to feedback effects; for example, shifting the relationship between water availability and transboundary governance indirectly influences the approach to infrastructure for resilience, which in turn influences water availability. In contrast, if natural infrastructure is assumed to be most effective for improving resilience, the collaborative response generates significant improvement in water availability and governance outcomes. Thus, collaboration—in combination with enhanced investment approaches, a collective and integrated data and knowledge system, and recognition of Indigenous water rights—can improve water availability.

The *true market for ecological goods and services* clearly increased the frequency of preferred outcomes in the scenario results (i.e., adequate water availability, improved water quality, improved ecological integrity). Additionally, several consistent scenarios flip from an intensive agricultural economy to regenerative agriculture and diversification. Importantly, this strategy created the least biased model prototype, in which no variant has a bias statistic under 10%. Thus, a true market for ecological goods and services partially decouples environmental and economic goals, creating an enabling environment for diversified and regenerative agriculture and desirable ecological outcomes. However, some consistent scenarios shift away from fully recognized Indigenous water rights, collaborative governance and collective data and knowledge systems. This unintended consequence is because under this version of the model, environmental outcomes are no longer contingent on an inclusive governance context. Thus, if not pursued carefully, a true market for ecological goods and services risks creating an environmentally desirable but socially undesirable system.

Few participants discussed *effective demand management,* but several consistent scenarios flipped toward improved water availability (e.g., chronically deficient to unequal; unequal to adequate). This finding reflects the direct impact of reducing anthropogenic pressure on water availability.

### Knowledge (re)integration: debrief workshop

The knowledge (re)integration phase was isolated to a simple knowledge sharing workshop (as discussed in “Knowledge (re)integration” section), which focused on translating model outputs into more accessible formats and stimulating discussion among participants. Five of the eight strictly robust consistent scenarios from Fig. 4 were selected for the debrief workshop to keep the number of scenarios manageable for participants. The scenarios were selected by including the most diverse scenarios in the set.

#### Narratives and visual art

**Fig. 6:**
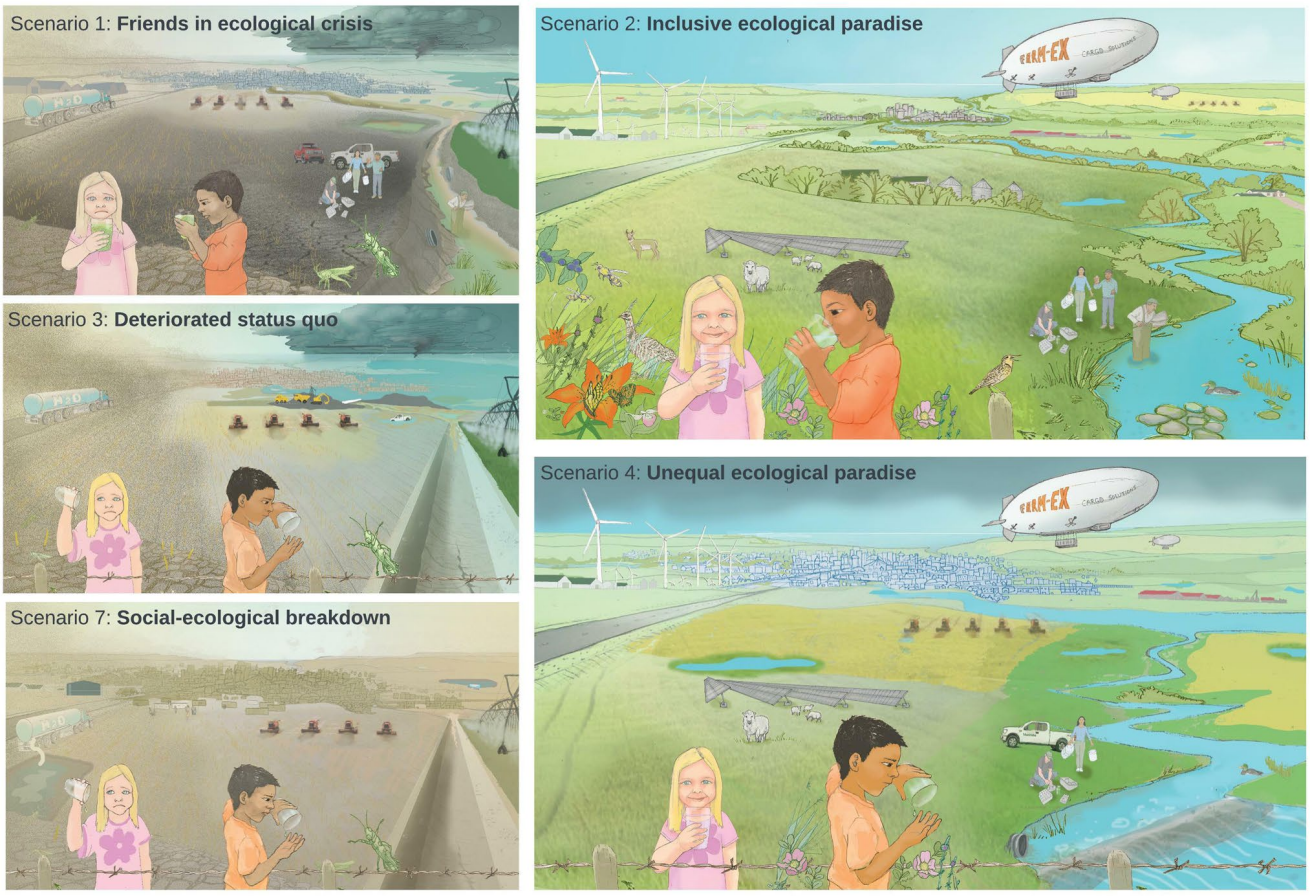
Artist visual depictions of five divergent robust scenarios for debrief workshop. Artist: Rhian Brynjolson

A local artist depicted the scenarios as visual art, involving two rounds of feedback with researchers. The artist depictions are shown in Fig. 6. The narratives for the five selected scenarios are included in S8.

#### Sharing and discussion

The workshop breakout sessions split the 22 participants in four breakout groups, each with four to six participants and a facilitator. During the desirability ranking exercise, participants agreed on the best- and worst-case scenarios and discussed implicit trade-offs. For example, participants discussed scenario 4 as desirable, but recognized it only seems to benefit those with power. Participants also recognized that the most desirable scenario (i.e., scenario 2) represented the most significant transformation from the status quo. Yet, participants in two groups stated that many aspects of scenario 2 are already occurring at small scales.

Participants agreed that the most plausible scenarios (by 2050) were those that do not depart significantly from the status quo. Different groups ranked different scenarios as most plausible (i.e., 1, 3, 4, and 7), surfacing important assumptions about the future. For example, participants thought scenarios containing the optimistic global climate change outcome are implausible, and that improved social outcomes (e.g., full recognition of Indigenous water rights) are more plausible than improved environmental outcomes (e.g., high ecological integrity). This confidence in the plausibility of full recognition of Indigenous water rights is notable, given its active role in realizing desirable scenarios (“Consistent scenarios and statistics” section).

Participants diverged more significantly in the discussion regarding how efforts to build resilience contribute to scenario outcomes. For example, one group thought a planned floodwater diversion scheme project promotes desirable outcomes for water availability (e.g., scenario 2), while the other discussed how such large-scale projects reinforce systems that are not resilient (e.g., scenario 3). This finding shows that participants not only hold divergent perspectives, but that participants may interpret scenarios according to their different views and interests.

The debrief at the end of the workshop revealed that the scenario process was valuable for three reasons: to make sense of complexity, surface different perspectives, and affirm the value of collaboration. Participant quotes supporting each of these statements are included in S9, but examples include:

“I don’t think it changed how I thought about the basin, but… the scenario approach is just so effective. It presents a range, and you sort of look at these different gradations along the continuum and I just think it’s an excellent, excellent way to consider during complex situations.”

“[We have] done a lot of work on kind of how decision-making is informed by both kind of facts and evidence but also perspectives… Being in these breakout groups is a good reminder that we all have priorities, biases, and just different places from which we’re coming to.”

“It was kind of a reminder that by bringing people together… around scenarios like this may actually change the way our future is shaped and how we prioritize.”

## Study implications and conclusion

We facilitated a transdisciplinary scenario modeling process in the RRB that explores big-picture scenarios of a river basin under climate change, characterizing future change as emergent from interactions between diverse efforts to build resilience and a complex, cross-scale SES. We used CIB to structure the process, a semi-quantitative scenario method that has been underutilized in SES research. In doing so, we also aimed to explore the potential for the CIB method to surface diverse perspectives and drivers of change in SESs. The resulting “big picture” scenarios reflect a more integrated and systemic picture than is offered by many quantitative scenario models and narrative scenario methods. To our knowledge, this is the first study to apply CIB in a participatory, transboundary context to explicitly characterize SES change, offering important implications for the RRB and sustainability research broadly.

### Implications for the Red River Basin

The study results surfaced three important implications for the RRB. First, the internally consistent scenarios depict multiple basins of attraction for the RRB. The scenarios integrate a wide range of drivers, from global agricultural markets to Indigenous water rights and water quality, and a broad scope of outcomes. The seed concept (i.e., small-scale, present innovations at scale) pushed governance descriptors toward more transformative scenarios. Actors may use these scenarios in strategy and policy making, pushing discussions toward a richer scope of outcomes than may otherwise be considered.

Second, the CIB matrix characterizes the RRB as a complex stability landscape of social–ecological interactions, exposing influential variables and feedbacks that affect the trajectory of the future. Actors may use the findings of the CIB analysis to enrich their understanding of the system, helping leverage cornerstone drivers of change (e.g., recognition of Indigenous water rights; culture and politics), situate solutions within a bigger picture of social–ecological interactions (e.g., collaborative governance only improves water availability if accompanied by a suite of other enabling governance conditions), and connect existing initiatives to their potential long-term implications (e.g., large-scale infrastructure contributes to resilience in complex and contested ways).

Finally, the sensitivity analysis generated scenarios that are robust to uncertainty yet revealed uncertainty and disagreement regarding how drivers of change interact. Actors may direct research efforts toward lesser understood but important interactions, such as between the state of the economy, culture and politics, and Indigenous governance. More targeted and integrative studies may analyze the systemic effects of diverse efforts to build resilience. In addition, participatory and deliberative spaces are required where actors can expose and discuss divergent perspectives and interests in resilience.

### Implications for sustainability science

The study offers important implications for sustainability science. First, the CIB method synthesized the expertise of diverse participants by integrating drivers of change represented by quantitative (e.g., water quality or climate) and qualitative knowledge (e.g., culture and politics), enabling the development of ‘big picture’ scenarios. Importantly, this integration process required a ‘meet in the middle’ approach so that direct interactions between drivers understood quantitatively versus qualitatively could be put on comparable footing and captured. In other words, deriving descriptors and variants from highly detailed quantitative studies sacrificed some degree of numerical granularity, while deriving descriptors and variants from qualitative theories and experiences sacrificed narrative richness. Moreover, the process of quantifying influence judgments helped make the assumptions about how such quantitative and qualitative descriptors interact explicit but were challenging to quantify in the matrix format of CIB (i.e., due to the indistinctness and ambiguity of such interactions, see “Influence judgments and multiple prototypes” section). Thus, our study demonstrated the opportunities and constraints in the ‘meet in the middle’ process required to apply this scenario modeling approach, which affirms the potential for and guides more widespread adoption of semi-quantitative scenario methods like CIB in the toolbox of SES modeling approaches.

Second, scenarios are often used to make the inherent unpredictability of SESs explicit, but the complexity of SES change means that there are significant gaps in the knowledge required to systematically model the future. Rather than setting rigid assumptions that reduce or ignore this uncertainty, our approach for sensitivity analysis (i.e., using multiple prototypes to identify scenarios that are ‘robust’ to these uncertainties) demonstrates one of multiple possible approaches to acknowledging and systematically embedding a wide range of uncertainties into the scenario process. We urge sustainability scientists using scenarios to draw from our experience to ensure scenario validity by addressing the full range of uncertainties (Dewulf and Biesbroek 2018) in a method- or context-specific and transparent way.

Third, our scenario development process was guided by a social–ecological scenario framework (i.e., described in “Social–ecological scenario framework” section), which was developed specifically to structure the development of big-picture scenarios in a river basin SES. This study thus offers a unique contribution to scenario practice in sustainability science by demonstrating the use of a guiding framework that is both theory- and method-informed (i.e., bringing together existing theory about the dynamics of SES change with the structure and capacities of the CIB method). This approach allowed for a systematic and transparent scenario analysis and helped leverage compatibilities between SES theory and the CIB method. Many scenario studies are guided by generic frameworks that are theory-informed but lack such a clear link to the unique capacities and limitations of the chosen scenario method. For example, STEEPV (social, technological, economic, environmental, political, and values) is used to maximize the scope of drivers (e.g., Proskuryakova 2022), or frameworks like Three Horizons are used to characterize transformation (Sharpe et al. 2016). Such frameworks are useful, but future studies can build on our findings by (1) adapting our ‘social–ecological scenario framework’ for applications of CIB in SES research and (2) developing new theory- and method-informed frameworks to guide scenario analysis, thereby adding further rigor to the use of scenarios in SES research and sustainability science more broadly.

Finally, sustainability scientists should reflect upon the ways in which, despite best efforts, every scenario process has limitations that excludes certain perspectives and drivers. In this study, focusing on robust scenarios may have masked divergent scenarios that are internally consistent only under marginalized assumptions. Similarly, the positionality of the researchers and the choice to avoid coding Indigenous knowledge limited the degree of Indigenous participation, inhibiting opportunities to generate scenarios that challenge dominant narratives. Further, the ‘meet in the middle’ approach required to formalize interviewee knowledge into a CIB model excluded Indigenous knowledge due to ethical concerns, and may favor scientific knowledge over local or practitioner knowledge given the academic bias to consult literature under uncertainty. Lastly, our study focused on a rigorous scenario development approach, but the final phase of ‘knowledge sharing’ lacked the deep collaboration required to fully reintegrate findings in the research context. Moreover, we did not use robust frameworks for evaluating learning through the process (Baird et al. 2014; Elsawah et al. 2020). Future applications of scenarios in sustainability science can draw from the limitations of our study to improve the rigor and impact of scenarios.

In sum, our analysis surfaced significant complexities surrounding efforts to build resilience and affirmed the potential for the CIB method to generate unique insights about the trajectory of SESs and opportunities for systemic interventions.

## Supplementary Information

Below is the link to the electronic supplementary material.

Supplementary file1 (DOCX 1710 KB)

## Data Availability

The authors confirm that relevant data to support the findings of this study are available within the article and its supplementary materials. The raw data from research participants (e.g., interview transcripts) are not publicly available, as they may contain information that could compromise the privacy of research participants.
