# Supplementary material for: Exploring “big picture” scenarios for resilience in social–ecological systems: transdisciplinary cross-impact balances modeling in the Red River Basin
Source: Sustain Sci. 2023 Apr 1;18(4):1773–94. doi: 10.1007/s11625-023-01308-1 (PMC10066973; doi:10.1007/s11625-023-01308-1)
Supplement: Supplementary file 1 — Supplementary file1 (DOCX 1710 KB) [file 11625_2023_1308_MOESM1_ESM.docx]

**S1 - ROUND 1 INTERVIEW PROTOCOL**

Part A: Participant information

1. How would you describe your expertise in the Red River Basin?
2. What is your current role and with what organization?

Part B: Scenarios Exogenous change

1. *What do you think are the **most important and uncertain external social future developments** influencing governance and management of the Red River Basin in the coming 30 years (to 2050)? Brainstorm and choose 3, using prompts on the Miro board if needed.
2. *What do you think are the **most important and uncertain external ECOLOGICAL/ ENVIRONMENTAL future developments** influencing governance and management of the Red River Basin in the coming 30 years? Brainstorm and choose 3, using prompts on the Miro board if needed.


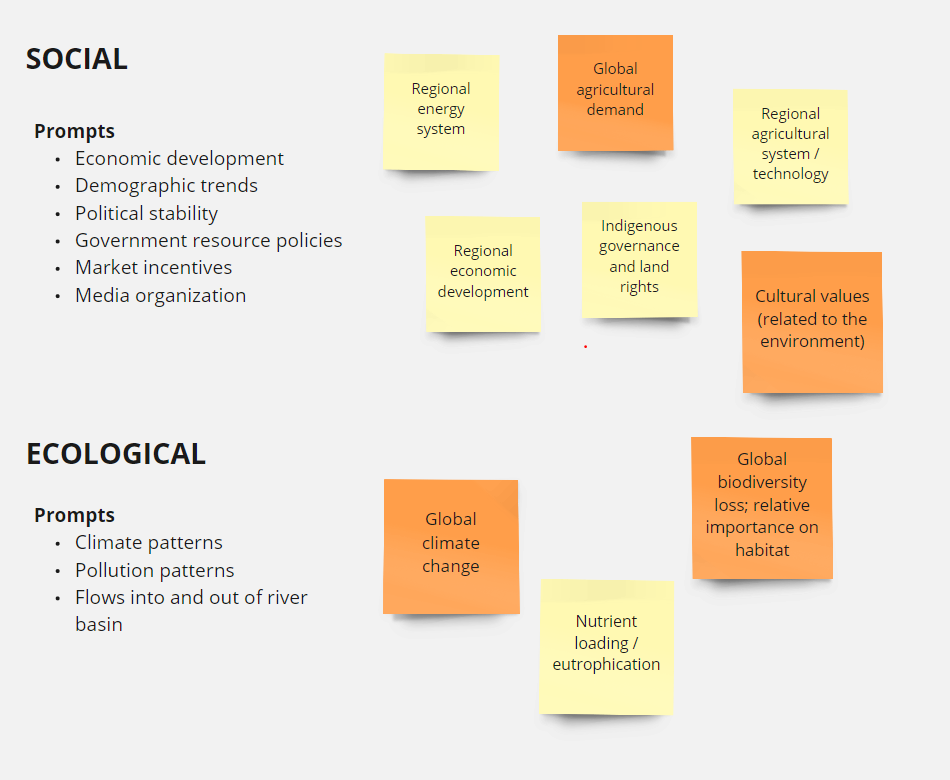


*Figure A: Screenshot of Miro template after one interview. The virtual sticky notes captured the most important and uncertain future developments discussed.*

1. *What **possible mutually exclusive end states** can you imagine for each of these social and ecological future developments in 30 years?


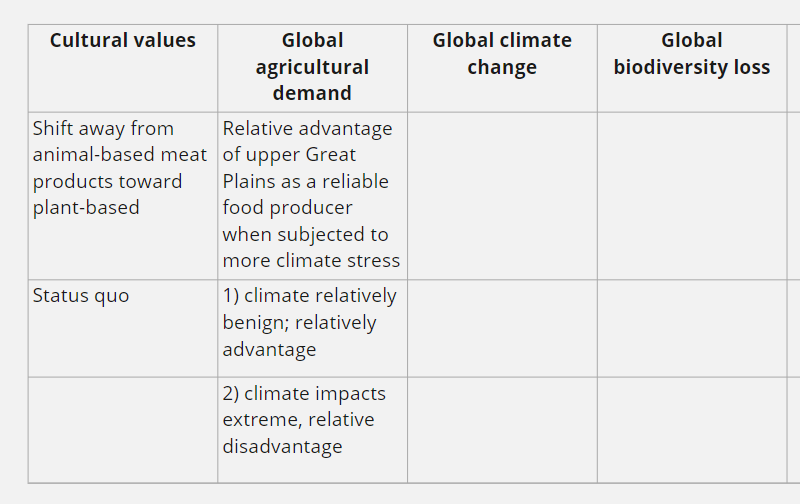


*Figure B: Screenshot of Miro template used to question 5. The future developments from questions 3 and 4 were put in the top row, with mutually exclusive end states discussed in the rows below.*

Part C: Resilience

*Defining resilience*

We often hear the term ‘resilience’ understood and interpreted in many ways, all describing strategies to cope with change and avoid transitioning to an undesirable state.

In our research we define resilience broadly to include all of these: the ability to withstand disturbance, the ability to recover from a disturbance, and to adapt and improve following a disturbance.

*Resilience to what?*

Resilience is usually determined in relation to a form of disturbance, some sort of long-term stressor or shock. In initial framings of this study, we were interested in resilience to increasing climate variability and change, primarily in the form of shocks like floods and droughts but also long-term stressors associated with changing seasonality and weather patterns.

1. What do you think are the most important impacts of existing and future climate variability in the Red River Basin (i.e., increasing frequency and severity of extreme floods and droughts)?

*Resilience over what time frame?*

We are looking at mid-century scenarios to 2050 in order to see how present-day resilience-building efforts will serve the Red River Basin of 2050.

*Resilience in the Red River Basin*

1. What does a resilient future look like to you?
2. What (potentially innovative) practices or projects are being pursued in the RRB that may contribute to resilience, in your view?
3. What features of the Red River Basin need to change to achieve this future?
4. What features need to be preserved to achieve this future? In other words, what existing aspects of the RRB contribute to resilience?

Optional questions (if not responded to, the responses to questions 8 – 11 will suffice):

1. *What do you think are the **most important and uncertain ECOLOGICAL/ENVIRONMENTAL future developments** relevant to resilience to climate variability in the Red River Basin in the coming 30 years? Brainstorm and then choose 3, using prompts if needed.
2. *What do you think are the **most important and uncertain SOCIAL future developments** relevant to resilience to climate variability in the Red River Basin in the coming 30 years? Brainstorm and then choose 3, using prompts if needed.


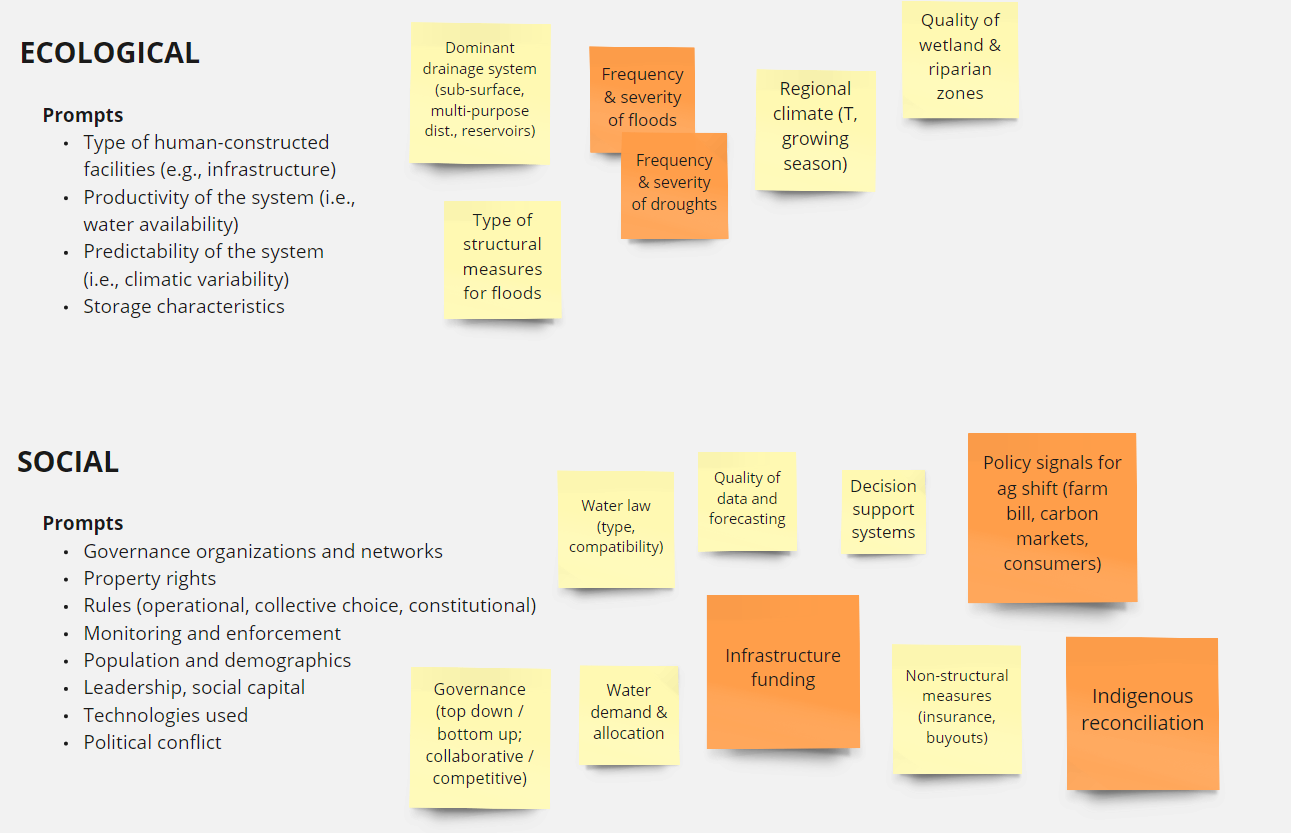


*Figure C: Screenshot of completed Miro template to support questions 11 and 12. The virtual sticky notes captured the key variables associated with resilience.*

1. *What **possible end states** can you imagine for each of these social and ecological future developments in 30 years (if time)?


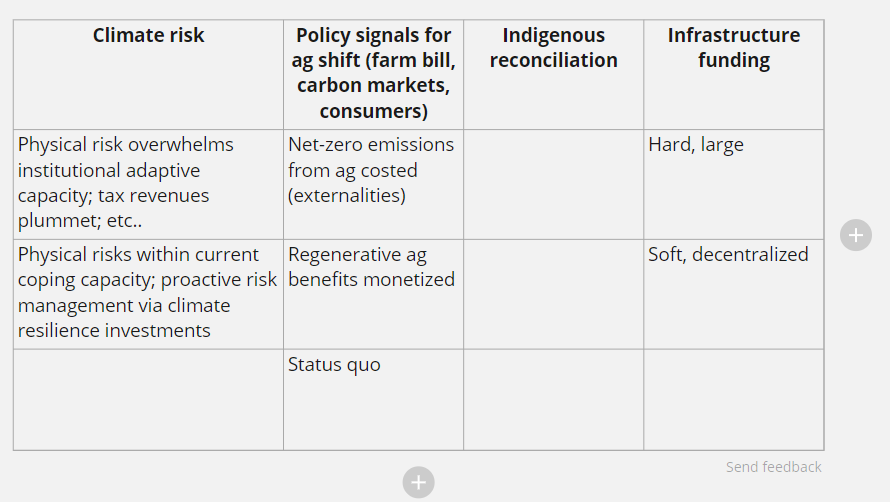


*Figure D: Screenshot of Miro template to support question 13. The variables from questions 11 and 12 were put in the top row, with mutually exclusive end states discussed in the rows below.*

**Closing script**

1. Can you recommend any other participants I should talk to? For each suggested participant ask for contact information or ask the interview if they would be willing to forward an invitation on my behalf.

Thank you so much for your responses to these questions. You will receive a thank you letter and eGift card shortly. With your permission I may contact you for the purposes of clarification, validation, and elaboration of your interview up to 3 weeks from today and once all stage 1 interviews are complete, I will send you a summary of the synthesis of stage 1 for your feedback. With your permission you may hear from me with an invitation for a follow up interview and/or workshop.

Thank you very much for participating in this study.

**S2 - ROUND 2 INTERVIEW PROTOCOL**

Participant information

1. How would you describe your expertise in the Red River Basin?
2. What is your current role and with what organization?

**Describe the interview purpose**

The method we are using to model these long-term scenarios is called cross-impact balances. This method describes the future as combinations of multiple end-states for different future developments. In round 1 interviews, we developed a list of multiple future social and environmental developments relevant for efforts to build resilience to climate variability and change in the Red River Basin. We determine multiple possible end states for each of those future developments in 2050. We also generated a list of interventions for resilience [describe how these are incorporated in the model; TBD based on the type of data collected in round 1 interviews].

In this interview, I will ask you a series of questions pertaining to your expertise on [insert relevant expertise]. The purpose of these questions is to determine the “influence judgments” between different end states of each uncertainty.

Do you have any questions?

STEP 1: Direct influences between social and environmental developments

The first step is determining direct influences between social and environmental developments identified in round 1 interviews. I will share a Miro board on my screen now to help us do so using a conceptual map. *share Miro board* We are going to draw arrows of direct influence between the future developments on this screen.

1. *Does future development X directly influence future development Y? [Repeat systematically one-by-one through each combination of social or ecological developments]


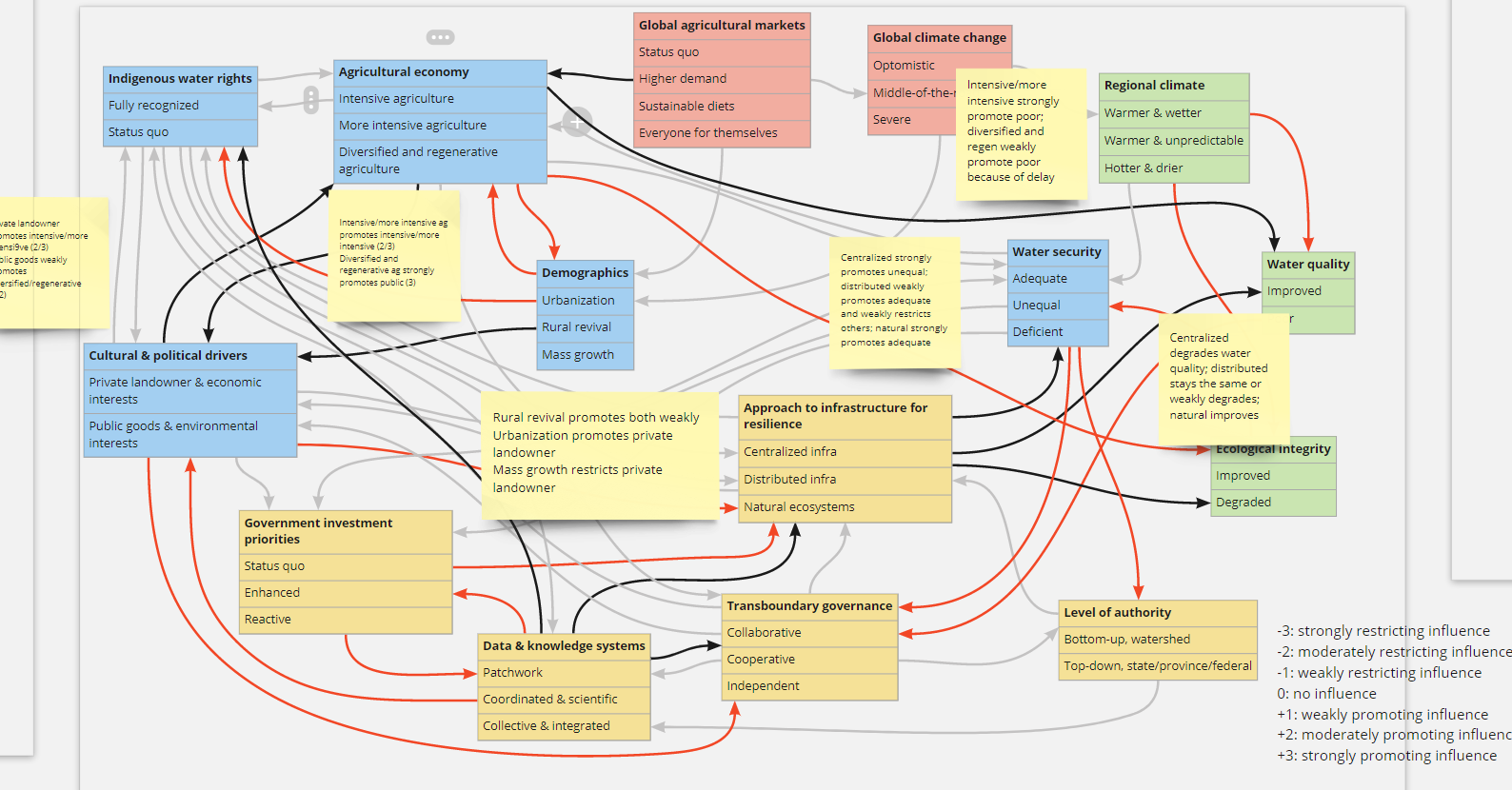


*Figure A: Screenshot of the Miro template after one of the interviews. The red arrows in the network diagram indicate the judgment sections that were both uncertain and relevant to the interviewee’s expertise. The light yellow sticky notes were filled out by the interviewer as the interviewee shared their rationale for a direction and strength of the influence.*

STEP 2: Influence judgements between states of social and environmental developments

In step 2, we are going to determine influence judgments between the mutually exclusive states of the future developments that we determined are directly related in step 1 These influence judgements are integers (i.e., 1, 2, 3) ranging as follows:

- -3: strongly restricting influence
- -2: moderately restricting influence
- -1: weakly restricting influence
- 0: no influence
- +1: weakly promoting influence
- +2: moderately promoting influence
- +3: strongly promoting influence

Moving one-by-one through each square of the matrix depicted in the Miro board that is relevant for each , ask the following:

1. *Suppose the only piece of information given about the future of the Red River Basin is that future development X has the state x_i_. Would you expect the direct influence of X on future development Y to be a hint that scenario factor Y has the state y_i_ (positive points), or as a hint that scenario factor Y does not have the state y_i_ (negative points)?

*Example for clarity.* Say two factors determined in step 1 interviews are A) **climate change** and B) **economic development**. Step 1 interviews will also determine factor states – i.e., 2 to 4 mutually exclusive possible end-states for each factor. For example, A) **climate change** could be *severe* (RCP8.5), *high* (RCP4.5) or *moderate* (RCP2.6), and B) **economic development** could be *growing, stagnant,* or *declining.* Question 3 asks experts to relate factor states to one another. So, in the wording of question 4 above, “suppose the only piece of information given about the future of the Red River Basin is that **climate change** is ***severe (RCP8.5)***. Would you expect the direct influence of **climate change** on **economic development** to be a hint that **economic development** is ***declining***, or as a hint that **economic development** is ***not declining***?

1. *What is the scale of that influence (-3 to +3)?

*Example for clarity.* Following from the example above, interviewees will be asked to rate the degree of influence according to a Likert scale from -3 to +3. For example, if **climate change** is ***severe (RCP8.5)*** and the expert indicates they expect this to be a hint that **economic development** is ***declining***, they will be then asked to rate this relationship as -1, -2, or -3.

Step 3: Influence judgements between resilience interventions

For each of the interventions for resilience, either:

1. *Repeat questions 3 and 4 for the components of the matrix relevant for resilience.

AND/OR

Imagine a future in which this resilience intervention is scaled and mainstream.

1. Which end states do you think would be impacted (promoted or discouraged) by this resilience intervention?
2. (optional) How do you think different end states would impact this resilience intervention?

*More details for clarity:* In step 1 interviews, participants identified innovative practices being pursued in the Red River Basin that may contribute to resilience, what features of the Red River Basin need to change to become resilient, and what features need to be preserved to become resilient. These will be represented in the CIB model as either alternative states for future developments in the CIB matrix or as external perturbations to the CIB matrix. For the former, these alternative factor states will be related to the others through influence judgements (-3 to +3) by repeating questions 3 and 4 above. For the latter, experts will be asked questions 7 and 8, which indicate which factor states would inform a perturbation/intervention analysis.

**Closing**

Thank you so much for your responses to these questions. You will receive a thank you letter and eGift card shortly. With your permission I may contact you for the purposes of clarification, validation, and elaboration of your interview up to 3 weeks from today and once all stage 2 interviews are complete, I will send you a summary of the synthesis of stage 2 interviews for your feedback.

In this study I am gathering a variety of perspectives. If there are major differences between your judgements today and those of others, I may invite you to a short workshop with other interviewees to discuss them, with your permission. With your permission you may hear from me with an invitation to later stages of the study

Thank you very much for participating in this study.

**S3 - INFLUENCE JUDGMENTS**

The tables below are judgment sections that summarize the influence judgments. In a judgment section, the variants in the row influence the variants in the column. The influence judgments are promoting (+) or inhibiting (-) on a scale of weak (1), moderate (2), or strong (3). According to the conventions of the CIB method, a row in a judgment section must sum to zero. The ‘rationale’ column summarizes the justification for the influence judgments in the row, as stated by interviews and/or in literature.

The primary sources of data for the influence judgments were the round 1 and 2 interviews. The ‘supported by’ column indicates the degree to which interviewee statements clearly supported the rationale for a given section. The check (✓) indicates that statements from at least two interviewees in round 1 interviews and/or at least one (expert) interviewee from round 2 interviewees support the rationale for the row. The question mark (?) indicates that interviewee statements may support the rationale for the row, but there remained a lack of clarity of disagreement among interviewees. The cross (🗶) indicates that interviewees did not offer statements about the row.

Literature was used to triangulate interviewee responses. Thus, the ‘supported by’ column also indicates whether literature supported the rationale offered by interviewees. The check (✓) indicates that at least one literature source clearly validated the stated rationale. The question mark (?) indicates that literature may validate the stated rationale, but there remained a lack of clarity (e.g., considering context-specific factors in the Red River Basin). The cross (🗶) indicates that literature did not validate the interviewee claims (e.g., because no literature was available).

The uncertain influence judgments were subject to a sensitivity analysis. In the ‘sensitivity analysis’ column, any non-zero influence judgments that are not subject to sensitivity analysis were marked ‘none’. Any non-zero influence judgments that are uncertain (i.e., due to lack of support from interviews and/or literature) were marked according to which type of sensitivity analysis they are subject to. Type I addresses new relationships not included in the baseline, Type II involves adjustments of the scores for certain non-zero relationships in the baseline, and Type III addresses combinations of different sensitivities. S4 describes the protocol for the sensitivity analysis in detail.

|  | | Global climate change | | | Rationale | Supported by | | Sensitivity analysis |
| --- | --- | --- | --- | --- | --- | --- | --- | --- |
|  |  | Optimistic | Moderate | Severe |  | Literature | Interviews |  |
| Global ag markets | Status quo | -1 | 2 | -1 | Status quo' global agricultural markets are associated with the shared socioeconomic pathway (SSP) that does not deviate significantly from historical trends (SSP2). SSP 2 is associated with moderate challenges with mitigation. Thus, 'status quo' agricultural markets influence global climate change toward a moderate outcome and restrict both optimistic and severe outcomes. | ✓ | N/A | None |
|  | Higher demand | -3 | 1 | 2 | Higher demand' global agricultural markets reflect the SSP describing a fossil-fuel driven economy (SSP5), which is associated with high challenges with mitigation. Thus, 'higher demand' agricultural markets influence global climate change toward a moderate or severe outcome and restrict an optimistic outcome. | ✓ | N/A | None |
|  | Sustainable diets | 3 | -1 | -2 | Sustainable diets' global agricultural markets reflect the SSP that is a sustainable future (SSP1), which is associated with low challenges with mitigation. Thus, 'sustainable diets' agricultural markets influence global climate change toward an optimistic outcome and restrict both moderate and severe outcomes. | ✓ | N/A | None |
|  | Everyone for themselves | -3 | 1 | 2 | Everyone for themselves' agricultural markets reflect the SSP that is a highly fragmented future (SSP3)m which is associated with high challenges with mitigation. Thus, 'everyone for themselves' global agricultural markets influence global climate change to a moderate or severe outcome and restricts an optimistic outcome. | ✓ | N/A | None |

|  | | | Rural ag economy | | | | | | Rationale | | Supported by | | | | Sensitivity analysis |
| --- | --- | --- | --- | --- | --- | --- | --- | --- | --- | --- | --- | --- | --- | --- | --- |
|  |  |  | Intensive ag | Diversified & regenerative | | |  | |  |  | Literature | | Interviews | |  |
| Global ag markets | Status quo | | 3 | -3 | | |  | | Status quo' global agricultural markets are associated with the shared socioeconomic pathway (SSP) that does not deviate significantly from historical trends (SSP2). SSP 2 is associated with moderate challenges with mitigation. Thus, 'status quo' agricultural markets influence global climate change toward a moderate outcome and restrict both optimistic and severe outcomes. | | ✓ | | ✓ | | None |
|  | Higher demand | | 3 | -3 | | |  | | Higher demand' global agricultural markets reflect the SSP describing a fossil-fuel driven economy (SSP5), which is associated with high challenges with mitigation. Thus, 'higher demand' agricultural markets influence global climate change toward a moderate or severe outcome and restrict an optimistic outcome. | | ✓ | | ✓ | | None |
|  | Sustainable diets | | -2 | 2 | | |  | | **Rationale 1:** Sustainable diets' global agricultural markets reflect the SSP that is a sustainable future (SSP1), which is associated with low challenges with mitigation. Thus, 'sustainable diets' agricultural markets influence global climate change toward an optimistic outcome and restrict both moderate and severe outcomes. | | ✓ | | ? | | **Rationale 1 included in baseline. Type II sensitivity analysis of rationale 2.** |
|  |  |  | 2 | -2 | | |  | | **Rationale 2:** Existing intensive multinationals would capture new demand presented by international market pressure. | | ✓ | | ? | |  |
|  | Everyone for themselves | | -2 | 2 | | |  | | **Rationale 1:** Everyone for themselves' agricultural markets reflect the SSP that is a highly fragmented future (SSP3)m which is associated with high challenges with mitigation. Thus, 'everyone for themselves' global agricultural markets influence global climate change to a moderate or severe outcome and restricts an optimistic outcome. | | ? | | ? | | **Excluded from baseline. Type I sensitivity analysis of both rationale 1 and 2.** |
|  |  |  | 2 | -2 | | |  | | **Rationale 2:** Greater pressure to produce for domestic self-sufficiency increases pressure to intensify | |  |  |  |  |  |
|  | | Regional demographics | | | | | | Rationale | | Supported by | | | | Sensitivity analysis | |
|  |  | Urbanization | | | Rural revival | Mass growth | |  |  | Literature | | Interviews | |  |  |
| Global climate change | Optimistic | 1 | | | 1 | -2 | | Optimistic climate change scenarios influence demographics away from mass growth, as climate change impacts will be less, and fewer people are motivated to migrate. | | ✓ | | ✓ | | None | |
|  | Moderate | 0 | | | 0 | 0 | | Influence too uncertain. Could be trends in any direction. | | - | | - | | N/A | |
|  | Severe | -1 | | | -2 | 3 | | Severe climate change scenarios result in significant climate change impacts, influencing people to migrate to regions of relative climate security like the Red River Basin. | | ✓ | | ✓ | | None | |

|  | | | Regional climate | | | | | | | Rationale | | Supported by | | | | Sensitivity analysis |
| --- | --- | --- | --- | --- | --- | --- | --- | --- | --- | --- | --- | --- | --- | --- | --- | --- |
|  |  |  | Warmer wetter | | Warmer extreme | | Hotter drier | | |  |  | Literature | | Interviews | |  |
| Global climate change | Optimistic | | 2 | | 1 | | -3 | | | Optimistic global climate scenarios lead to some degree of warming by 2050 due to locked in emissions but avoid major and more extreme climatic shift. Increasing temperature leads to increased atmospheric moisture content, which increases average annual precipitation. | | ✓ | | ✓ | | None |
|  | Moderate | | -3 | | 2 | | 1 | | | Moderate global climate scenarios influence the regional climate toward a more extreme and unpredictable temperature & precipitation regime in the RRB. Higher chance of heat waves, heavy precipitation events, etc. Moderate global climate scenarios influence away from warmer & wetter outcomes as climate shifts outside of normal range of variability. Still potential to have more extreme hot and dry scenarios. | | ✓ | | ✓ | | None |
|  | Severe | | -3 | | 1 | | 2 | | | Severe global climate change outcomes influence the regional climate of the RRB toward a hotter and drier scenario. This will occur particularly if climatic tipping points are crossed and/or storm tracks shift. Potential still to have a hotter climate with more extreme variability (i.e., avoiding severe drought outcomes). | | ✓ | | ✓ | | None |
|  | | | | Reg. cultural & political drivers | | | | | Rationale | | Supported by | | | | Sensitivity analysis | |
|  |  |  |  | Private | | Public | |  |  |  | Literature | | Interviews | |  |  |
| Regional demographics | | Urbanization | | -2 | | 2 | |  | **Rationale 1:** Urban politics tend toward more progressive politics, which often emphasize environmental issues. Fewer farmers with traditionally greater focus on private landowner and economic interests. | | ✓ | | ✓ | | **Excluded from baseline. Type I sensitivity analysis of rationale 1 and 2.** | |
|  |  |  |  | 2 | | -2 | |  | **Rationale 2:** Urbanization takes more people out of rural areas, so people will not bear witness or mitigate land degradation, reinforcing a paradigm of private landowner and economic interests. | | ✓ | | ✓ | |  |  |
|  |  | Rural revival | | 2 | | -2 | |  | **Rationale 1:** Rural revival brings more city dwellers closer to the agricultural economy, so they will empathize more with challenges of growing food on private land and adopt private landowner & economic interests. Assumed influence to public goods rural demographic change would be minor in comparison. | | ✓ | | ✓ | | **Excluded from baseline. Type I sensitivity analysis of rationale 1 and 2.** | |
|  |  |  |  | -2 | | 2 | |  | **Rationale 2:** Rural citizens tend to act more on environmental stewardship as they are closer to nature. More rural citizens will bring more people into this culture, moderately promoting public goods and environmental interests. | | ✓ | | ✓ | |  |  |
|  |  | Mass growth | | 0 | | 0 | |  | Mass growth may influence both private and public values, depending on the demographic characteristics of those migrating to the region. | | - | | - | | N/A | |

|  | | Rural ag economy | | | Rationale | Supported by | | Sensitivity analysis |
| --- | --- | --- | --- | --- | --- | --- | --- | --- |
|  |  | Intensive ag | Diversified & regenerative ag |  |  | Literature | Interviews |  |
| Regional demographics | Urbanization | 3 | -3 |  | Urbanization leaves fewer people in rural areas with diverse skills for the labour force, further entrenching existing intensive economic system. High urbanization rates encroach on agricultural land, intensifying land that already exists as ag. Assumed increased urban demand would not push for greater diversification. | ✓ | ✓ | Supported by interviews and literature. |
|  | Rural revival | -2 | 2 |  | Rural revival brings more people with diverse skills to rural areas, offering opportunities for diversified economy. More people in rural areas to buy local products will increase demand for more diverse products. Assumption that there is no increase in competition for land for housing, etc. | ? | ✓ | **Excluded from baseline. Type I sensitivity analysis.**  *Uncertainty regarding whether this relationship holds under 21st century conditions (e.g., automation).* |
|  | Mass growth | -2 | 2 |  | **Rationale 1:** Mass growth will bring more people with diverse skills and needs to both urban and rural areas, offering opportunity and demand for diversified economy. | ? | ✓ | **Excluded from baseline. Type I sensitivity analysis for rationale 1 and 2.** |
|  |  | 1 | -1 |  | **Rationale 2:** Questions remain about how many people and where they are housed, and associated implications on land pressure. Potential weak link to more intensive as available ag land is pressured to produce more. |  |  |  |

|  | | Water security | | | Rationale | Supported by | | Sensitivity analysis |
| --- | --- | --- | --- | --- | --- | --- | --- | --- |
|  |  | Adequate | Unequal | Deficient |  | Literature | Interviews |  |
| Regional demographics | Urbanization | -1 | 2 | -1 | Urbanization reduces rural water demand but puts pressure on existing urban water resources, potentially driving unequal outcomes between rural and urban areas | ✓ | ✓ | None |
|  | Rural revival | -2 | 1 | 1 | Challenge with rural outmigration tied to associated agricultural activity. Potential for unequal or deficient outcomes. | ✓ | ✓ | None |
|  | Mass growth | -3 | 1 | 2 | Pop growth in both rural and urban areas increases demand and associated economic activity across the board, strongly increasing the risk of deficiency | ✓ | ✓ | None |

|  | | Gov investment priorities | | | Rationale | Supported by | | Sensitivity analysis |
| --- | --- | --- | --- | --- | --- | --- | --- | --- |
|  |  | Status quo | Flexible | Reactive |  | Literature | Interviews |  |
| Regional demographics | Urbanization | -1 | -1 | 2 | Sparse populations in rural areas have challenges attracting government funds (i.e., more investment per person, so disincentive for government involvement), potentially contributing to a reactive approach. | 🗶 | ✓ | **Excluded from baseline. Type I sensitivity analysis.** |
|  | Rural revival | 0 | 0 | 0 | No direct influence | - | - | N/A |
|  | Mass growth | 0 | 0 | 0 | No direct influence | - | - | N/A |

|  | | Rural ag economy | | | Rationale | Supported by | | Sensitivity analysis |
| --- | --- | --- | --- | --- | --- | --- | --- | --- |
|  |  | Intensive ag | Diversified regenerative |  |  | Literature | Interviews |  |
| Reg cultural & politics | Private | 3 | -3 |  | Private landowner and economic interests reinforce existing intensive agriculture or motivate more intensive agriculture, limiting regenerative agriculture or diversification due to longer-term, more uncertain economic benefits. | ✓ | ✓ | None. |
|  | Public | -2 | 2 |  | Environmental values moderately promote regenerative/organic agriculture for its sustainability benefits. For example, if people are paying farmers for wetland restoration and ecosystem benefits, or if farmers choose to take action themselves due to their own value system. | ✓ | ✓ | None. |

|  | | Transboundary governance | | | Rationale | Supported by | | Sensitivity analysis |
| --- | --- | --- | --- | --- | --- | --- | --- | --- |
|  |  | Collaborative | Cooperative | Independent |  | Literature | Interviews |  |
| Reg cultural & politics | Private | -3 | 1 | 2 | Private cultural values impede collaborative decision making, as diverse interests and needs are evaluated against profit maximization and individual land owner priorities. | 🗶 | ✓ | **Excluded from baseline. Type I sensitivity analysis.** |
|  | Public | 2 | 1 | -3 | Public cultural values influence a shift away from highly independent decision making, as public goods are shared and require greater collaboration or cooperation to meaningfully address. | 🗶 | ✓ | **Excluded from baseline. Type I sensitivity analysis.** |

|  | | Infra resilience | | | Rationale | Supported by | | Sensitivity analysis |
| --- | --- | --- | --- | --- | --- | --- | --- | --- |
|  |  | Centralized | Distributed infra | Natural |  | Literature | Interviews |  |
| Reg cultural & politics | Private | 3 | -1 | -2 | Private values influence uptake of centralized or distributed infrastructure, which are highly managed and can offer near-term economic gains. Disincentive to preserve wetlands if they are not compensated equal to agricultural profits. Possible restricting of distributed as would require many landowners to contribute a portion of productive land. | ✓ | ✓ | None. |
|  | Public | -3 | 1 | 2 | Public values influence natural or distributed infrastructure outcomes, since they offer clearer benefits to the environment (e.g., valuing ecosystem services) | ✓ | ✓ | None. |

|  | | Government investment approach | | | Rationale | Supported by | | Sensitivity analysis |
| --- | --- | --- | --- | --- | --- | --- | --- | --- |
|  |  | Conventional | Enhanced | Reactive |  | Literature | Interviews |  |
| Reg cultural & politics | Private | 1 | -3 | 2 | Private economic & landowner interests have a moderate positive influence on reactive and status quo approach. Across the basin, landowners are highly involved in local and regional government and if they believe there is nothing wrong with the way things are then they will want to stay the same (i.e., status quo and reactive). | 🗶 | ✓ | **Excluded from baseline. Type I sensitivity analysis.** |
|  | Public | -3 | 2 | 1 | Public goods and environmental interests have a moderate positive influence on the enhanced approach, but would also weakly promote reactive because emergency measures may always be needed and depend primarily on other factors. | 🗶 | ✓ | **Excluded from baseline. Type I sensitivity analysis.** |

|  | | Indigenous water rights | | | Rationale | Supported by | | Sensitivity analysis |
| --- | --- | --- | --- | --- | --- | --- | --- | --- |
|  |  | Fully recognized | Status quo |  |  | Literature | Interviews |  |
| Reg cultural & politics | Private | -2 | 2 |  | Private landowner and economic interests driving decision making would negatively influence further recognition of Indigenous values and water rights, reinforcing the status quo. | ✓ | ✓ | None. |
|  | Public | 2 | -2 |  | Public goods and environmental interests are more compatible with Indigenous relationships to the land, so would moderately influence further recognition of Indigenous values and water rights. | ✓ | ✓ | None. |

|  | | Regional demographics | | | Rationale | Supported by | | Sensitivity analysis |
| --- | --- | --- | --- | --- | --- | --- | --- | --- |
|  |  | Urbanization | Rural revival | Mass growth |  | Literature | Interviews |  |
| Rural ag economy | Intensive ag | 2 | -2 | 0 | Intensive agriculture leads to more centralization, with agglomeration in larger farming organizations. This trend has caused the existing trend of urbanization over the last century. Assumed no direct influence on mass growth | ✓ | ✓ | None. |
|  | Diversified & regen ag | -3 | 3 | 0 | Diversification would strongly promote rural revival and reduce urbanization, because there will be more amenities and diverse jobs available. Assumed no direct influence on mass growth. | ✓ | ✓ | None. |

|  | | Reg. cultural & political drivers | | | Rationale | Supported by | | Sensitivity analysis |
| --- | --- | --- | --- | --- | --- | --- | --- | --- |
|  |  | Private | Public |  |  | Literature | Interviews |  |
| Rural ag economy | Intensive ag | 3 | -3 |  | Intensive agriculture tends to be necessarily more extractive to get a return on your investment. When agriculture has a more hierarchical structure, there is conventionally less interest in the labour and inputs that goes into food and more interest in private landowner and economic interests. This is a reciprocal, self-reinforcing relationship. | ✓ | ✓ | None. |
|  | Diversified & regen ag | -2 | 2 |  | The relationship between the type of rural economy and cultural values was weak historically (i.e., influenced primarily by other factors), but this link may be stronger now. Farmers values are oriented toward wanting to do something about climate change, sustainability, etc. but currently do not see an economically viable pathway. | ✓ | ✓ | None. |

|  | | Water availability | | | Rationale | Supported by | | Sensitivity analysis |
| --- | --- | --- | --- | --- | --- | --- | --- | --- |
|  |  | Adequate | Unequal | Deficient |  | Literature | Interviews |  |
| Rural ag economy | Intensive ag | -2 | 1 | 1 | Intensive agriculture has significant water demand if driven by irrigation, and in the RRB agriculture is prioritized over other sectors potentially driving inequalities. Also, intensive agriculture perpetuates a drainage culture that moves water off the landscape and downstream quickly, potentially reducing water availability in times of scarcity. | ✓ | ✓ | None. |
|  | Diversified & regen ag | 3 | -1 | -2 | Diversified and regenerative agriculture distributes water demands across different products and sectors. Also, regenerative agriculture includes perennial crops and improves soil health, which retains more moisture across the landscape. | ✓ | ✓ | None. |

|  | | Water quality | | Rationale | Supported by | | Sensitivity analysis |
| --- | --- | --- | --- | --- | --- | --- | --- |
|  |  | Improved | Poor |  | Literature | Interviews |  |
| Rural ag economy | Intensive ag | -3 | 3 | Intensive agriculture influences water quality to be poor due to chemical and nutrient inputs onto the landscape that are washed into water bodies. | ✓ | ✓ | None. |
|  | Diversified & regen ag | 1 | -1 | Shift toward diversified & regenerative agriculture improves water quality due to decreased nutrients and chemical inputs. Additionally, improved soil health obtained through regenerative agricultural practices is expected to reduce erosion, improve water retention, etc. Some evidence that improved crop diversity would also reduce nitrogen and phosphorous leaching. Improvement to water quality has a slow response time, so significant improvements may not be seen on the landscape before 2050, even if practices changed quickly in the 2020s. | ✓ | ✓ | None. |

|  | | Ecological integrity | |  | Rationale | Supported by | | Sensitivity analysis |
| --- | --- | --- | --- | --- | --- | --- | --- | --- |
|  |  | High | Low |  |  | Literature | Interviews |  |
| Rural ag economy | Intensive ag | -3 | 3 |  | Intensive agriculture promotes ecological degradation primarily due to land disturbance and pollution. | ✓ | ✓ | None. |
|  | Diversified & regen ag | 3 | -3 |  | Regenerative agriculture promotes higher ecological integrity due to improvement of ecosystem services and promotion of biodiversity and wildlife habitat. | ✓ | ✓ | None. |

|  | | Infra resilience | | | Rationale | Supported by | | Sensitivity analysis |
| --- | --- | --- | --- | --- | --- | --- | --- | --- |
|  |  | Centralized | Distributed infra | Natural |  | Literature | Interviews |  |
| Rural ag economy | Intensive ag | 1 | 1 | -2 | Intensive agriculture influences approach to infra resilience to be centralized or distributed, as it requires drainage systems that are highly managed and controlled. | ? | ✓ | **Excluded from baseline. Type I sensitivity analysis.**  *In addition to lack of validation in literature, aspects of this influence are indirect and reinforce the relationship between cultural & political drivers and the approach to infrastructure for resilience.* |
|  | Diversified & regen ag | -2 | 1 | 1 | Diversified and regenerative ag economy influences approach to infra resilience to be natural or distributed to service a wider range of economic needs. | 🗶 | ✓ | **Excluded from baseline. Type I sensitivity analysis.**  *In addition to lack of validation in literature, aspects of this influence are indirect and reinforce the relationship between cultural & political drivers and the approach to infrastructure for resilience.* |

|  | | Rural ag economy | | | Rationale | Supported by | | Sensitivity analysis |
| --- | --- | --- | --- | --- | --- | --- | --- | --- |
|  |  | Intensive ag | Diversified & regenerative ag |  |  | Literature | Interviews |  |
| Water availability | Adequate | 2 | -2 |  | **Rationale 1:** Adequate water availability allows for continuation of status quo (intensive) or opportunities to become more water intensive. No influence to shift toward a more regenerative agricultural system. | 🗶 | 🗶 | **Excluded from baseline. Type I sensitivity analysis of rationale 1 and 2** |
|  |  | 1 | -1 |  | **Rationale 2:** Adequate water availability allows for continuation of status quo (intensive) or opportunities to become more water intensive. No influence to shift toward a more regenerative agricultural system. | 🗶 | 🗶 |  |
|  | Unequal | -1 | 1 |  | **Rationale 1:** Unequal water availability may reduce viability of intensive agriculture, particularly if it requires irrigation. Potential incentive to shift toward diversification/regenerative ag. | ? | ✓ | **Excluded from baseline. Type I sensitivity analysis of rationale 1 and 2.** |
|  |  | 2 | -2 |  | **Rationale 2:** Unequal water availability results in some closure of smaller farms, resulting in further consolidation in intensive agriculture. Only weakly promoting more intensive agriculture as water allocations may be restricted. | 🗶 | ✓ |  |
|  | Deficient | -2 | 2 |  | **Rationale 1:** Intensive agriculture is only possible with sufficient water availability, including irrigation under drought scenarios. Deficiency reduces the viability of this type of system and may influence a shift in the system toward regenerative ag. | ? | ✓ | **Excluded from baseline. Type I sensitivity analysis of rationale 1 and 2.** |
|  |  | 3 | -3 |  | **Rationale 2:** Deficient water availability leads to mass closures of smaller farms, dramatically consolidating and entrenching the intensive agriculture system. | 🗶 | ✓ |  |

|  | | Transboundary governance | | | Rationale | Supported by | | Sensitivity analysis |
| --- | --- | --- | --- | --- | --- | --- | --- | --- |
|  |  | Collaborative | Cooperative | Independent |  | Literature | Interviews |  |
| Water availability | Adequate | 2 | 1 | -3 | **Rationale 1:** Adequate water availability encourages meaningful collaborative or cooperative relationships between jurisdictions. | 🗶 | ✓ | **Excluded from baseline. Type I sensitivity analysis of rationale 1 and 2.** |
|  |  | -3 | 1 | 2 | **Rationale 2:** Adequate water security offers no incentive for jurisdictions to work together, so it would inhibit collaboration and promote independence. There is always some degree of cooperation. | 🗶 | ✓ |  |
|  | Unequal | -1 | -1 | 2 | **Rationale 1:** Unequal water availability strains collaborative and cooperative governance arrangements, as jurisdictions attempt to protect their own resources, at the expense of others. | ✓ | ✓ | **Rationale 1 included in baseline. Type II sensitivity analysis of rationale 2.** |
|  |  | 1 | 1 | -2 | **Rationale 2:** Unequal outcomes would bring people together to solve issues collectively, driving collaboration or cooperation. | 🗶 | ✓ |  |
|  | Deficient | -2 | -1 | 3 | **Rationale 1:** Deficient water availability creates conflict and breaks down cooperation and collaboration. | ✓ | ✓ | **Rationale 1 included in baseline. Type II sensitivity analysis of rationale 2.** |
|  |  | 2 | 1 | -3 | **Rationale 2:** Deficiency would drive a collaborative approach. After a single event, people might move toward an independent approach, but chronic deficiency over time would bring jurisdictions together. | 🗶 | ✓ |  |

|  | | Gov investment priorities | | | Rationale | Supported by | | Sensitivity analysis |
| --- | --- | --- | --- | --- | --- | --- | --- | --- |
|  |  | Conventional | Flexible | Reactive |  | Literature | Interviews |  |
| Water availability | Adequate | 2 | -1 | -1 | Adequate water availability reinforces a conventional government investment approach, as there is no incentive to pivot toward other approaches. | 🗶 | ✓ | **Excluded from baseline. Type I sensitivity analysis.** |
|  | Unequal | 1 | -3 | 2 | Unequal water availability would promote a reactive or status quo approach, because of the need for crisis management. Government would only be motivated to adopt an enhanced approach if the lack of availability is chronic and money must pour into the system. | 🗶 | ✓ | **Excluded from baseline. Type I sensitivity analysis.** |
|  | Deficient | -3 | 2 | 1 | Chronic deficiency would force governments to react with an enhanced approach. Still reactive approach would coexist because of need to support near-term issues. | 🗶 | ✓ | **Excluded from baseline. Type I sensitivity analysis.** |

|  | | Authority | | | Rationale | Supported by | | Sensitivity analysis |
| --- | --- | --- | --- | --- | --- | --- | --- | --- |
|  |  | Local | Top-down |  |  | Literature | Interviews |  |
| Water availability | Adequate | 2 | -2 |  | Adequate water availability encourages local, bottom-up governance | 🗶 | ✓ | **Excluded from baseline. Type I sensitivity analysis.** |
|  | Unequal | -2 | 2 |  | Unequal water availability causes conflict, encouraging more top-down, command-and-control governance | 🗶 | ✓ | **Excluded from baseline. Type I sensitivity analysis.** |
|  | Deficient | -2 | 2 |  | Deficient water availability causes loss, damage, and conflict, encouraging "states of emergency" and top-down, command-and-control governance. | 🗶 | ✓ | **Excluded from baseline. Type I sensitivity analysis.** |

|  | | Approach to infra for resilience | | | Rationale | Supported by | | Sensitivity analysis |
| --- | --- | --- | --- | --- | --- | --- | --- | --- |
|  |  | Centralized | Distributed | Natural |  | Literature | Interviews |  |
| Indigenous water rights | Fully recognized | -3 | 1 | 2 | Fully recognized Indigenous water rights and more prominent Indigenous values would positively influence an approach to infrastructure for resilience that prioritizes natural ecosystems and a whole-system (or distributed) approach. Additionally, it would promote a shift *away* from a centralized system, which results in winners and losers and conventionally protects urban areas and prominent economic sectors over Indigenous land and communities. | ✓ | ✓ | None. |
|  | Status quo | 2 | 1 | -3 | Continued status quo (meaning lack of) role of Indigenous values and water rights in decision making would moderately reinforce the centralized system and significantly de-prioritize natural ecosystems. These were assumed based on the inverse of the rationale articulated above. | 🗶 | 🗶 | **Excluded from baseline. Type I sensitivity analysis.** |

|  | | Cultural & political drivers | | | Rationale | Supported by | | Sensitivity analysis |
| --- | --- | --- | --- | --- | --- | --- | --- | --- |
|  |  | Private | Public |  |  | Literature | Interviews |  |
| Indigenous water rights | Fully recognized | -3 | 3 |  | Fully recognized Indigenous water rights would imply more prominent Indigenous values in the culture and politics driving decision making. This would hint toward a culture that prioritizes public goods and the environment over private landowner and economic interests when required, as this better reflects Indigenous relationships to the land. | ✓ | ✓ | None. |
|  | Status quo | 2 | -2 |  | Inverse of rationale above. Status quo (meaning lack of) role of Indigenous values and water rights in water-related decision making moderately reinforces the existing support for private landowner and economic interests. | ✓ | ✓ | None. |

|  | | Rural economy | | | Rationale | Supported by | | Sensitivity analysis |
| --- | --- | --- | --- | --- | --- | --- | --- | --- |
|  |  | Intensive | Diversified & regenerative |  |  | Literature | Interviews |  |
| Indigenous water rights | Fully recognized | -2 | 2 |  | Resource intensity and ecological degradation driven by intensive agriculture is generally not compatible with an Indigenous worldview. Fully recognized Indigenous land and water rights would moderately influence a shift toward a diversified and regenerative agricultural economy. | ✓ | ✓ | None. |
|  | Status quo | 0 | 0 |  | No direct influence. | - | - |  |

|  | | Data & Knowledge Systems | | | Rationale | Supported by | | Sensitivity analysis |
| --- | --- | --- | --- | --- | --- | --- | --- | --- |
|  |  | Patchwork | Coordinated & scientific | Collaborative & integrated |  | Literature | Interviews |  |
| Indigenous water rights | Fully recognized | 0 | -3 | 3 | Fully recognized Indigenous values and water rights would strongly influence preference for a collaborative and integrated data and knowledge system over a coordinated & scientific knoweldge system, due to the strengthened role of Indigenous and local knowledge holders in decision making. Assumed no influence on patchwork data collection, because it would be influenced by other factors. | ✓ | ✓ | None. |
|  | Status quo | 0 | 2 | -2 | The status quo moderately influences preference for coordinated & scientific data and knowledge systems and restricts collaborative & integrated knowledge systems, because Indigenous and local knowledge holders' role in decision making is not prevalent. Assumed no influence on patchwork data collection, because it would be influenced by other factors. | ✓ | ? | None. |

|  | | Transboundary governance | | | Rationale | Supported by | | Sensitivity analysis |
| --- | --- | --- | --- | --- | --- | --- | --- | --- |
|  |  | Collaborative | Cooperative | Independent |  | Literature | Interviews |  |
| Indigenous water rights | Fully recognized | 3 | -1 | -2 | Fully recognized Indigenous water rights would require high degrees of collaboration to resolve challenges associated with allocation, etc. | ✓ | ✓ | None. |
|  | Status quo | -2 | 1 | 1 | Status quo governance lacks common goals and meaningful involvement of Indigenous communities in governance and decision making. Status quo reflects historical exclusion of Indigenous communities from transboundary decision making. | ✓ | ✓ | None. |

|  | | Water security | | | Rationale | Supported by | | Sensitivity analysis |
| --- | --- | --- | --- | --- | --- | --- | --- | --- |
|  |  | Adequate | Unequal | Deficient |  | Literature | Interviews |  |
| Regional climate | Warmer & wetter | 3 | -1 | -2 | Warmer and wetter climate promotes adequate water availability, as extremes are manageable and within the range of variability buffered by existing infrastructure. | ✓ | ✓ | None. |
|  | Warmer & extreme | -3 | 2 | 1 | Warmer & extreme climate encourages promotes unequal availability over time and space due to hydroclimatic uncertainty beyond extremes buffered by existing infrastructure. | ✓ | ✓ | None. |
|  | Hotter & drier | -3 | 1 | 2 | Hotter & drier climate encourages deficient water availability. | ✓ | ✓ | None. |

|  | | Water quality | | | Rationale | Supported by | | Sensitivity analysis |
| --- | --- | --- | --- | --- | --- | --- | --- | --- |
|  |  | Improved | Poor |  |  | Literature | Interviews |  |
| Regional climate | Warmer & wetter | -2 | 2 |  | **Rationale 1:** An abundance of water would flush contaminants into water bodies, both during snowmelt and rainfall events. This would increase the load of contaminants in the Red River system. | ✓ | ✓ | **Excluded from baseline. Type I sensitivity analysis with both rationale 1 and 2.** |
|  |  | 1 | -1 |  | **Rationale 2:** An abundance of water would flush contaminants into water bodies, but it would also reduce their concentration and residence time, which would slightly improve the water quality. | ✓ | ✓ |  |
|  | Warmer & extreme | -3 | 3 |  | More extreme variability would flush more contaminants into water bodies during peak flow events. Any buildup of contaminants on the landscape during low-flow or drought events would also be flushed into the system. This would increase the load of contaminants in the Red River system. | ✓ | ✓ | None. |
|  | Hotter & drier | -2 | 2 |  | **Rationale 1:** A hotter and drier climate sees less water on the surface, which means poorer quality in terms of temperature, concentration of contaminants, etc. Even under a hotter & drier scenario there will still be rainfall, and because the Prairies are continental these rainfall events will likely be more extreme. | ✓ | ✓ | **Rationale 1 included in baseline. Type II sensitivity of rationale 2.** |
|  | Hotter & drier | 1 | -1 |  | **Rationale 2:** A hotter and drier reduces landscape runoff, so less contaminants are drawn into the Red River system. | ✓ | ✓ |  |

|  | | Ecological integrity | | | Rationale | Supported by | | Sensitivity analysis |
| --- | --- | --- | --- | --- | --- | --- | --- | --- |
|  |  | High | Low |  |  | Literature | Interviews |  |
| Regional climate | Warmer & wetter | 1 | -1 |  | Overall, a warmer & wetter climate would improve ecological integrity because you're increasing ecological activity by adding more moisture to the environment and avoiding severe dry spells. This influence is weak because it is tempered by the higher temperature, which changes the type of organisms that will thrive in the ecosystem. | ? | ✓ | **Excluded from baseline. Type I sensitivity analysis.**  *Uncertainty remains regarding whether a warmer & wetter climate would improve ecological integrity (e.g., by introducing more moisture and biological activity) or would decrease ecological integrity due to warmer temperatures that shift the climate regime away from its "natural state".* |
|  | Warmer & extreme | -1 | 1 |  | Warmer & more extreme variability reduces ecological integrity, because the ecosystems are always trying to bounce back from the more recent dry spells. This influence is weak because it is tempered by the positive role of disturbance in facilitating ecological activity. | ✓ | ✓ | None. |
|  | Hotter & drier | -2 | 2 |  | Hotter & drier climate promotes low ecological integrity because availability of water is a key indicator of survivability as ecosystems are constantly recovering from severe dry periods. Also, the temperature regime is extended beyond organisms' preferred range. | ✓ | ✓ | None. |

|  | | Ecological integrity | | | Rationale | Supported by | | Sensitivity analysis |
| --- | --- | --- | --- | --- | --- | --- | --- | --- |
|  |  | High | Low |  |  | Literature | Interviews |  |
| Water quality | Improved | 3 | -3 |  | Improved water quality improves ecological integrity, for example as eutrophication and algal blooms are diminished due to reduced nutrient loading. | ✓ | ✓ | None. |
|  | Poor | -3 | 3 |  | Poor water quality exacerbates ecological degradation. For example, nutrient loading contributes to eutrophication and algal blooms and poor water quality affects benthic integrity. | ✓ | ✓ | None. |

|  | | Water quality | | | Rationale | Supported by | | Sensitivity analysis |
| --- | --- | --- | --- | --- | --- | --- | --- | --- |
|  |  | Improved | Poor |  |  | Literature | Interviews |  |
| Ecological integrity | High | 2 | -2 |  | Higher ecological integrity improves the capacity of the system to naturally capture and treat contaminants. Lower risk of algal blooms, etc. | ✓ | ✓ | None. |
|  | Low | -2 | 2 |  | Inverse rationale as above | ✓ | ✓ | None. |

|  | | Water quality | | | Rationale | Supported by | | Sensitivity analysis |
| --- | --- | --- | --- | --- | --- | --- | --- | --- |
|  |  | Improved | Poor |  |  | Literature | Interviews |  |
| Transboundary governance | Collaborative | 3 | -3 |  | A collaborative approach with common goal and understanding among jurisdictions would significantly improve water quality by resolving the upstream-downstream dynamic. | 🗶 | ✓ | **Excluded from baseline. Type I sensitivity analysis.** |
|  | Cooperative | 1 | -1 |  | Cooperation between institutions helps negotiate between multiple interests in efforts to improve water quality. Still limited by locked-in perspectives and individual goals limit significant improvements. | ? | ✓ | **Excluded from baseline. Type I sensitivity analysis.**  *Uncertainty due to history of cooperative commitments to improving water quality yet there is a lack of evidence that they have improved water quality.* |
|  | Independent | -2 | 2 |  | Tension and locked-in perspectives between jurisdictions restrict improvement to water quality, because it is difficult to motivate voluntary commitments | 🗶 | ✓ | **Excluded from baseline. Type I sensitivity analysis.**  *Uncertainty due to history of cooperative commitments to improving water quality yet there is a lack of evidence that they have improved water quality.* |

|  | | Data systems | | | Rationale | Supported by | | Sensitivity analysis |
| --- | --- | --- | --- | --- | --- | --- | --- | --- |
|  |  | Patchwork | Coordinated and scientific | Collective & integrated |  | Literature | Interviews |  |
| Transboundary gov | Collaborative | -3 | 1 | 2 | Collaborative governance meaningfully engages with all jurisdictions and perspectives at all levels, including Indigenous. Would be conducive environment for a more integrated and collective approach to data and modelling. | ✓ | ✓ | None. |
|  | Cooperative | 1 | 2 | -3 | Cooperative governance still driven by independent goals, encouraging coordinated/scientific for better data coverage, but lacks common goals and Indigenous engagement for truly collective/integrated. | ✓ | ✓ | None. |
|  | Independent | 3 | -1 | -2 | Independent governance strongly influences a patchwork approach, as each jurisdiction pursues data collection independently. Lack of structure for coordination or collective action. | ✓ | ✓ | None. |

|  | | Infra resilience | | | Rationale | Supported by | | Sensitivity analysis |
| --- | --- | --- | --- | --- | --- | --- | --- | --- |
|  |  | Centralized | Distributed infra | Natural |  | Literature | Interviews |  |
| Transboundary gov | Collaborative | -2 | 1 | 1 | Collaborative governance supports approaches that benefit the whole system, rather than individual needs. Positive influence toward distributed and natural approaches as demonstrated by existing bottom-up initiatives. | 🗶 | ✓ | **Excluded from baseline. Type I sensitivity analysis** |
|  | Cooperative | 0 | 0 | 0 | Cooperative governance would support all three types, so no direct influence to any. | 🗶 | ✓ | None. |
|  | Independent | 2 | -1 | -1 | Independent governance encourages centralized infra, as it helps secure individual needs (e.g., major diversions), sometimes at the expense of the whole. | ✓ | ✓ | None. |

|  | | Authority | | | Rationale | Supported by | | Sensitivity analysis |
| --- | --- | --- | --- | --- | --- | --- | --- | --- |
|  |  | Local | Top-down |  |  | Literature | Interviews |  |
| Transboundary gov | Collaborative | 3 | -3 |  | Collaborative governance connects bottom-up initiatives to a larger whole, empowering learning, etc. | 🗶 | ✓ | **Excluded from baseline. Type I sensitivity analysis.** |
|  | Cooperative | 0 | 0 |  | No clear direct influence | - | - | None. |
|  | Independent | -3 | 3 |  | Independent governance creates potential for conflict/tensions that lead to more top-down control. | ? | ✓ | **Excluded from baseline. Type I sensitivity analysis.** |

|  | | Indigenous water rights | | | Rationale | Supported by | | Sensitivity analysis |
| --- | --- | --- | --- | --- | --- | --- | --- | --- |
|  |  | Fully recognized | Status quo |  |  | Literature | Interviews |  |
| Transboundary gov | Collaborative | 2 | -2 |  | Assumption that transboundary governance that meaningfully includes Indigenous communities and leaders in decision making would have a weak to moderate influence on Indigenous values and water rights becoming more prominent. | ✓ | ✓ | None. |
|  | Cooperative | 0 | 0 |  | No direct influence. | - | - | None. |
|  | Independent | 0 | 0 |  | No direct influence. | - | - | None. |

|  | | Gov investment priorities | | | Rationale | Supported by | | Sensitivity analysis |
| --- | --- | --- | --- | --- | --- | --- | --- | --- |
|  |  | Conventional | Enhanced | Reactive |  | Literature | Interviews |  |
| Data systems | Patchwork | 1 | -2 | 1 | Lack of (updated) information makes governments more reactive or lets them fall back on proven solutions with value that is proven and easy-to-understand. | 🗶 | ✓ | **Excluded from baseline. Type I sensitivity analysis.** |
|  | Coordinated and scientific | 1 | 2 | -2 | More coordinated scientific data also reveals cost of inaction (reactive). While more holistic scientific data may clarify the value of both status quo and flexible investments, it may not reveal the hidden costs of status quo options. | ✓ | ✓ | **Excluded from baseline. Type I sensitivity analysis.** |
|  | Collective & integrated | -1 | 3 | -2 | More holistic approach to data collection helps reveal cost of inaction (i.e., the reactive investment approach) and the hidden costs of status quo investments. This would encourage a shift toward more flexible options. | 🗶 | ✓ | **Excluded from baseline. Type I sensitivity analysis.** |

|  | | Infra resilience | | | Rationale | Supported by | | Sensitivity analysis |
| --- | --- | --- | --- | --- | --- | --- | --- | --- |
|  |  | Centralized | Distributed infra | Natural |  | Literature | Interviews |  |
| Data systems | Patchwork | 3 | -2 | -1 | **Rationale 1:** If data systems are patchwork, decision makers will not know what is happening on the landscape and try to "control" uncertainty and resort to conventional investments in centralized infrastructure. Assumption that distributed infrastructure is more restricted than natural. | ✓ | ✓ | **Rationale 1 included in baseline. Type II sensitivity analysis of rationale 2.** |
|  |  | 3 | -1 | -2 | **Rationale 2:** Same as rationale 1, but assumed that distributed infrastructure is more restricted than natural. | ✓ | ✓ |  |
|  | Coordinated and scientific | -3 | 2 | 1 | Coordinated and scientific data systems help see the systems as a whole, enabling a distributed, watershed-based approach to managing water. Relying on natural ecosystems also involved some degree of control, which may also be enabled by coordinated and scientific data systems. | ✓ | ✓ | None. |
|  | Collective & integrated | -3 | 1 | 2 | Collective and integrated data system enables design and coordination of a distributed system. Because local and Indigenous knowledges are also included, would more strongly support natural ecosystems approach. Also, reveals hidden costs of centralized system (e.g., diverting problems elsewhere; downstream impacts). | ✓ | ✓ | None. |

|  | | Water quality | | | Rationale | Supported by | | Sensitivity analysis |
| --- | --- | --- | --- | --- | --- | --- | --- | --- |
|  |  | Improved | Poor |  |  | Literature | Interviews |  |
| Data & knowledge | Patchwork | -1 | 1 |  | Data & knowledge systems allow for targeted water quality interventions. Patchwork data systems contribute to lack of knowledge regarding where and what interventions will work. | 🗶 | ✓ | **Excluded from baseline. Type I sensitivity analysis.** |
|  | Cooperative & scientific | 1 | -1 |  | Cooperative & scientific data and knowledge system allow for targeted interventions to improve water quality. | 🗶 | ✓ | **Excluded from baseline. Type I sensitivity analysis.** |
|  | Collective & integrated | 1 | -1 |  | Collective & integrated data and knowledge system allow for targeted interventions to improve water quality. | 🗶 | ✓ | **Excluded from baseline. Type I sensitivity analysis.** |

|  | | Indigenous water rights | | | Rationale | Supported by | | Sensitivity analysis |
| --- | --- | --- | --- | --- | --- | --- | --- | --- |
|  |  | Fully recognized | Status quo |  |  | Literature | Interviews |  |
| Data & knowledge system | Patchwork | 0 | 0 |  | No direct influence. | - | - | None. |
|  | Coordinated & scientific | 0 | 0 |  | No direct influence. | - | - | None. |
|  | Collective & integrated | 1 | -1 |  | Collective and integrated data and knowledge would include Indigenous voices, contributing to further empowerment of Indigenous interests in water-related decision making. This interaction is reciprocal, and stronger in the opposite direction. | ✓ | ✓ | None. |

|  | | Transboundary | | | Rationale | Supported by | | Sensitivity analysis |
| --- | --- | --- | --- | --- | --- | --- | --- | --- |
|  |  | Collaborative | Cooperative | Independent |  | Literature | Interviews |  |
| Data & knowledge system | Patchwork | -1 | -1 | 2 | Lack of data acts as a key barrier to more cooperative and collaborative forms of governance. | ✓ | ? | None. |
|  | Coordinated & scientific | 0 | 0 | 0 | No direct influence. | - | - | None. |
|  | Collective & integrated | 2 | -1 | -1 | A collective & integrated data and knowledge system would reinforce a collaborative governance approach. This interaction is reciprocal and stronger in the opposite direction. | ✓ | ? | None. |

|  | | Data systems | | |  | Supported by | | Sensitivity analysis |
| --- | --- | --- | --- | --- | --- | --- | --- | --- |
|  |  | Patchwork | Coordinated and scientific | Collective & integrated | Rationale | Literature | Interviews |  |
| Gov investment approach | Conventional | 1 | 2 | -2 | Status quo investment priorities encourage either patchwork systems (as currently exist) or investment in coordinated/scientific data systems required to calculate status quo ROIs, etc. | 🗶 | ✓ | **Excluded from baseline. Type I sensitivity analysis.** |
|  | Enhanced | -3 | 1 | 2 | Flexible investment priorities encourage coordinated or collective data systems, as it encourages a more proactive approach and investment in soft infrastructure | 🗶 | ✓ | **Excluded from baseline. Type I sensitivity analysis.** |
|  | Reactive | 3 | -1 | -2 | Reactive investment priorities encourages a patchwork data system, as the alternatives require a longer-term, pro-active view | 🗶 | ✓ | **Type I sensitivity analysis.** |

|  | | Infra resilience | | | Rationale | Supported by | | Sensitivity analysis |
| --- | --- | --- | --- | --- | --- | --- | --- | --- |
|  |  | Centralized | Distributed infra | Natural |  | Literature | Interviews |  |
| Gov investment approach | Conventional | 3 | -1 | -2 | Status quo government investment priorities encourage centralized infra systems, as large-scale, centralized systems cater to traditional investment tools/assessment techniques and do not similarly value other forms of investment  **Included all stated influence judgments in this section in prototype 0.** | ✓ | ✓ | None. |
|  | Enhanced | -3 | 2 | 1 | **Rationale 1:** Flexible government investment priorities encourage distributed and natural systems, as these systems may be more economically efficient than centralized but difficult to finance under status quo priorities. Assumption that distributed will be more strongly promoted than natural infrastructure. | ✓ | ✓ | **Rationale 1 included in baseline. Type II sensitivity analysis of rationale 2.** |
|  |  | -3 | 1 | 2 | **Rationale 2:** Same as rationale 1. Assumption that natural will be natural more strongly than distributed infrastructure. |  |  |  |
|  | Reactive | 3 | -2 | -1 | **Rationale 1:** Reactive investment priorities reinforce centralized infra systems; difficult to move toward alternatives like distributed/natural. Assumption that distributed is more strongly restricted than natural. | 🗶 | ✓ | **Rationale 1 included in baseline. Type II sensitivity analysis of rationale 2.** |
|  |  | 3 | -1 | -2 | **Rationale 2:** Same as rationale 1. Assumption that natural is more strongly restricted than distributed. |  |  |  |

|  | | Water security | | | Rationale | Supported by | | Sensitivity analysis |
| --- | --- | --- | --- | --- | --- | --- | --- | --- |
|  |  | Adequate | Unequal | Deficient |  | Literature | Interviews |  |
| Infra resilience | Centralized | 3 | -1 | -2 | **Rationale 1:** Centralized infrastructure protects against major deficiency like the 1930s drought or worse, but storing water and redirecting it as needed during flood events. Centralized infrastructure avoids devastating outcomes from either floods or droughts and balances out unequal competition. | ✓ | ✓ | **Excluded from baseline. Type III sensitivity analysis of rationales 1,2, and 3 (i.e., rationale 1 for influence of centralized, distributed, and natural on water security in combination, etc.)** |
|  |  | -3 | 2 | 1 | **Rationale 2:** Centralized infra systems push problems elsewhere, perpetuating unequal access. Uncontrolled drainage reduces resilience to drought/low-flow periods, promoting deficient outcomes. | ✓ | ✓ |  |
|  |  | -3 | 2 | 1 | **Rationale 3:** Centralized infrastructure systems promotes unequal security because there will be winners and losers. Uncontrolled drainage reduces resilience to drought/low-flow periods. | ✓ | ✓ |  |
|  | Distributed infra | -1 | 2 | -1 | **Rationale 1:** Distributed infrastructure would provide some buffer against extremes but would ultimately promote an unequal outcome because it wouldn't have enough capacity for peak extremes. | ? | ✓ | **Excluded from baseline. Type III sensitivity analysis of rationales 1,2, and 3 (i.e., rationale 1 for influence of centralized, distributed, and natural on water security in combination, etc.)** |
|  |  | 3 | -2 | -1 | **Rationale 2:** Distributed infra systems retain water on the land where it is needed, helping reduce unequal access (problem not pushed away) and improving resilience to low-flow | ? | ✓ |  |
|  |  | 2 | -1 | -1 | **Rationale 3:** Distributed system would weakly or moderately improve water security, as it reduces landscape runoff. | ✓ | ✓ |  |
|  | Natural | -3 | 1 | 2 | **Rationale 1:** We know nature gives us ecosystem services and some resilience to drought through flood storage. However, it would lead to deficient water availability as it wouldn't handle the peaks. | ? | ✓ | **Excluded from baseline. Type III sensitivity analysis of rationales 1,2, and 3 (i.e., rationale 1 for influence of centralized, distributed, and natural on water security in combination, etc.)** |
|  |  | -2 | 1 | 1 | **Rationale 2:** Natural ecosystems inhibit adequacy because hydroclimatic variability exceeds natural capacity to buffer. | ? | ✓ |  |
|  |  | 3 | -1 | -2 | **Rationale 3:** Natural infra systems retain water on the land and built an even stronger natural buffer to low flow by improving capacity of soil to retain moisture. Many things would have to change for this to be in place by 2050, but if it were that would strongly promote adequate. The system has been so modified, so if you were able to restore some ecosystem functions that would be positive. | ✓ | ✓ |  |

|  | | Water quality | |  | Rationale | Supported by | | Sensitivity analysis |
| --- | --- | --- | --- | --- | --- | --- | --- | --- |
|  |  | Improved | Poor |  |  | Literature | Interviews |  |
| Infra resilience | Centralized | -2 | 2 |  | Centralized infra perpetuates uncontrolled drainage culture, which causes contaminants to migrate more easily from land to waterways. | ✓ | ✓ | None. |
|  | Distributed infra | 2 | -2 |  | Distributed infra helps retain water where it lands, reducing upstream-downstream runoff. Ability to optimize the system to meet various water quantity and quality objectives. | ✓ | ✓ | None. |
|  | Natural | 2 | -2 |  | **Rationale 1:** Wetlands and habitat provide water purification service. | ? | ✓ | **Rationale 1 included in baseline. Type II sensitivity analysis of rationale 2.** |
|  |  | -1 | 1 |  | **Rationale 2:** Natural systems may not perform their functions, e.g., due to context-specific factors, such as whether the nutrient loading reaches a critical threshold beyond which the wetland cannot handle, or if a wetland is not managed to ensure plants that release nutrient uptake back into the system are removed seasonally. |  |  |  |

|  | | Ecological integrity | |  | Rationale | Supported by | | Sensitivity analysis |
| --- | --- | --- | --- | --- | --- | --- | --- | --- |
|  |  | High | Low |  |  | Literature | Interviews |  |
| Infra resilience | Centralized | -2 | 2 |  | Centralized systems negatively impact ecological inteigrty by disrupting natural flows and draining wetlands. Inter-basin transfers risk introducing new potentially harmful biota to the Red River Basin system. | ✓ | ✓ | None. |
|  | Distributed infra | -1 | 1 |  | Distributed infrastructure creates opportunities to restore natural flow regimes and for habitat to develop around ponds. and reduce degradation from centralized infra systems. However, this influence is tempered by lack of priority to restore and protect diverse natural habitats, including different types of natural wetlands, etc. Also, reduces ecological integrity because there are more interventions on more landscape. | ? | ✓ | **Excluded from baseline. Type I sensitivity analysis.** |
|  | Natural | 2 | -2 |  | Natural ecosystem approach directly improves ecological integrity by prioritizing habitat restoration and associated ecosystem goods and services. | ✓ | ✓ | None. |

|  | | Data systems | | | Rationale | Supported by | | Sensitivity analysis |
| --- | --- | --- | --- | --- | --- | --- | --- | --- |
|  |  | Patchwork | Coordinated scientific | Collective integrated |  | Literature | Interviews |  |
| Authority | Local | 1 | -2 | 1 | Local, bottom-up authority encourages collective and integrated data systems as local/Indigenous/scientific knowledge is utilized as needed to solve local challenges (e.g., via citizen science). Potential for patchwork due to lack of top-down coordination. | ✓ | ✓ | None. |
|  | Top-down | 1 | 1 | -2 | Top-down authority encourages coordinated, scientific data to diagnose top-down solutions or patchwork data due to reluctance to disclose data. | ✓ | ✓ | None. |

|  | | Infra resilience | | | Rationale | Supported by | | Sensitivity analysis |
| --- | --- | --- | --- | --- | --- | --- | --- | --- |
|  |  | Centralized | Distributed infra | Natural |  | Literature | Interviews |  |
| Authority | Local | -3 | 2 | 1 | Local, bottom-up authority encourages natural and distributed infra, as water is managed where it lands and local stakeholders are aware of ecosystem services of distributed or natural systems. | 🗶 | ✓ | **Excluded from baseline. Type I sensitivity analysis.** |
|  | Top-down | 2 | 1 | -3 | Top-down authority includes centralized infra, as it is more conducive to a command-and-control approach. Highly managed distributed system may also be compatible with a distributed system. | 🗶 | ✓ | **Excluded from baseline. Type I sensitivity analysis.** |

**S4 – SENSITIVITY ANALYSIS**

The influence judgments described in Appendix C were subject to a detailed sensitivity analysis. First, prototype 0 includes only the influence judgments that are 1) not subject to sensitivity analysis (i.e., are not uncertain), or 2) are the better supported rationale (i.e., by interviews and literature) for judgment sections subject to type II sensitivity analysis (i.e., adjustments to non-zero influence judgments). All remaining judgment sections were uncertain and were zeroed, including those subject to type I (i.e., introductions of new influence judgments) or type III (i.e., combinations of sensitivities). The cross-impact matrix for Prototype 0 is depicted in Figure D1.

**
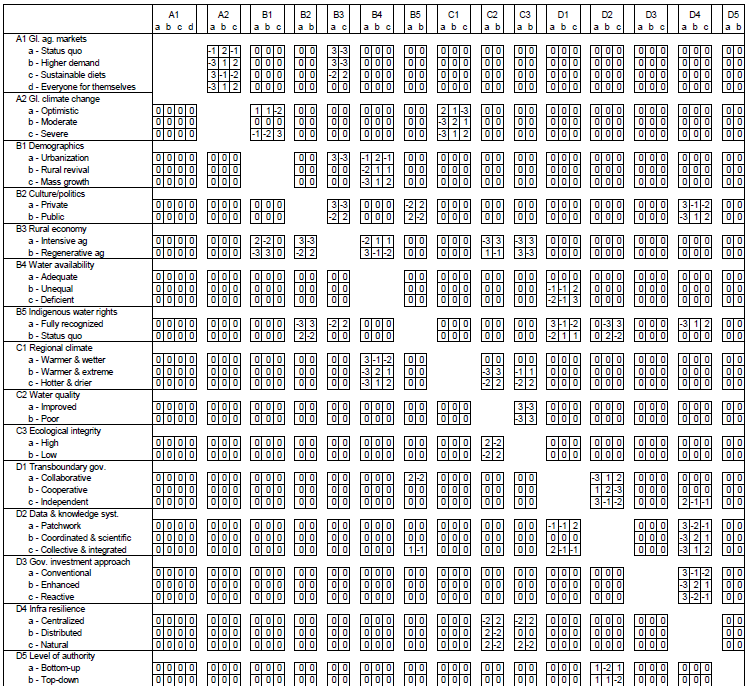
**

Figure D1: Summary of prototypes in sensitivity analysis

Prototype 0a, 0b, and 0c are differentiated by a Type III sensitivity on the influence of the ‘approach to infrastructure for resilience’ on ‘water availability’. This Type III sensitivity was structured into three versions of the judgment section, all of which were voiced by multiple participants but none of which could be validated with literature. Prototypes 1 (a, b, c) and 2 (a, b, c) include maximally diverse combinations of Type I and II sensitivity analyses as depicted in Figure D.2. where prototype 2 (a, b, c) both further augments the scope of uncertainty covered in prototypes 1 (a, b, c). Thus, the sensitivity analysis produced nine prototypes of the model. However, only the six prototypes of 1 (a, b, c) and 2 (a, b, c) were analyzed. Prototypes 0a, 0b, and 0c were not analyzed further: the results would not be meaningful given the number of uncertain judgment sections that were zeroed.


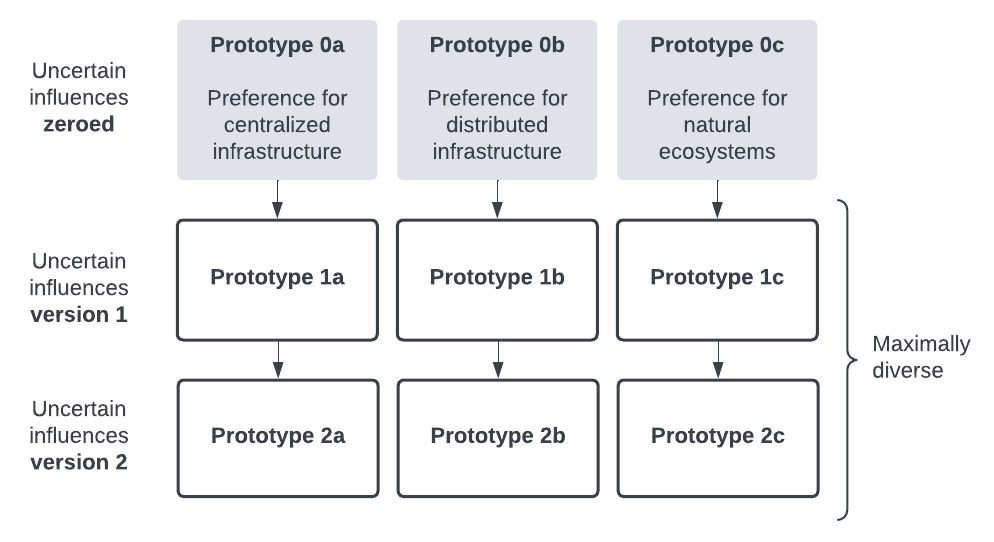


Figure D2: Summary of prototypes in sensitivity analysis

Table D1: Detailed description of each prototype with associated changes from prototype 0

| *Prototype* | | *Purpose* | *Changes from prototype 0* |
| --- | --- | --- | --- |
| 0 | a | Evaluate the Type III sensitivity analysis of the influence of ‘approach to infrastructure for resilience’ on ‘water availability’ with the assumption that centralized infrastructure is most effective for improving water availability. | \|  \| Adequate \| Unequal \| Deficient \| \| --- \| --- \| --- \| --- \| \| Centralized \| 3 \| -2 \| -1 \| \| Distributed \| -1 \| 2 \| -1 \| \| Natural \| -2 \| -1 \| 3 \| |
|  | b | Type III sensitivity analysis of the influence of ‘approach to infrastructure for resilience’ on ‘water availability’ with the assumption that distributed infrastructure is most effective for improving water availability. | \|  \| Adequate \| Unequal \| Deficient \| \| --- \| --- \| --- \| --- \| \| Centralized \| -1 \| 2 \| -1 \| \| Distributed \| 2 \| -1 \| -1 \| \| Natural \| -2 \| 2 \| 0 \| |
|  | c | Type III sensitivity analysis of the influence of ‘approach to infrastructure for resilience’ on ‘water availability’ with the assumption that natural ecosystems are most effective for improving water availability. | \|  \| Adequate \| Unequal \| Deficient \| \| --- \| --- \| --- \| --- \| \| Centralized \| -1 \| 2 \| -1 \| \| Distributed \| 2 \| -1 \| -1 \| \| Natural \| 3 \| -2 \| -1 \| |
| 1 | a  b  c | Evaluate all Type I and II sensitivities. | Transboundary governance 🡪 Water quality   \|  \| Improved \| Poor \| \| --- \| --- \| --- \| \| Collaborative \| 3 \| -3 \| \| Cooperative \| 1 \| -1 \| \| Independent \| -2 \| 2 \|   Transboundary governance 🡪 Approach to infra for resilience   \|  \| Centralized \| Distributed \| Natural \| \| --- \| --- \| --- \| --- \| \| Collaborative \| -2 \| 1 \| 1 \| \| Cooperative \| 0 \| 0 \| 0 \|   Transboundary governance 🡪 Level of authority   \|  \| Bottom-up \| Top-down \| \| --- \| --- \| --- \| \| Collaborative \| 3 \| -3 \| \| Cooperative \| 0 \| 0 \| \| Independent \| -3 \| 3 \|   Data systems 🡪 Government investment approach   \|  \| Conventional \| Enhanced \| Reactive \| \| --- \| --- \| --- \| --- \| \| Patchwork \| 1 \| -2 \| 1 \| \| Coordinated & scientific \| 1 \| 2 \| -3 \| \| Collective & integrated \| -1 \| 3 \| -2 \|   Data systems 🡪 Water quality   \|  \| Improved \| Poor \| \| --- \| --- \| --- \| \| Patchwork \| -1 \| 1 \| \| Cooperative & scientific \| 1 \| -1 \| \| Collective & integrated \| 1 \| -1 \|   Government investment approach 🡪 Data system   \|  \| Patchwork \| Coordinated & scientific \| Collective & integrated \| \| --- \| --- \| --- \| --- \| \| Conventional \| 1 \| 2 \| -2 \| \| Enhanced \| -3 \| 1 \| 2 \| \| Reactive \| 3 \| -1 \| -2 \|   Approach to infra for resilience 🡪 Ecological integrity   \|  \| High \| Low \| \| --- \| --- \| --- \| \| Distributed \| -1 \| 1 \|   Authority 🡪 Approach to infra for resilience   \|  \| Centralized \| Distributed \| Natural \| \| --- \| --- \| --- \| --- \| \| Bottom-up \| -3 \| 2 \| 1 \| \| Top-down \| 2 \| 1 \| -3 \| |
| 2 | a  b  c | Evaluate alternative rationales for Type I and II sensitivities. | Global agricultural markets 🡪 Rural economy   \|  \| Intensive agriculture \| Diversified & regenerative agriculture \| \| --- \| --- \| --- \| \| Everyone for themselves \| 2 \| -2 \|   Regional climate 🡪 Water quality   \|  \| Improved \| Poor \| \| --- \| --- \| --- \| \| Warmer & wetter \| 1 \| -1 \| \| Hotter & drier \| 1 \| -1 \|   Water availability 🡪 Rural economy   \|  \| Intensive agriculture \| Diversified & regenerative agriculture \| \| --- \| --- \| --- \| \| Adequate \| 1 \| -1 \| \| Unequal \| 2 \| -2 \| \| Deficient \| 3 \| -3 \|   Demographics 🡪 Cultural and political drivers   \|  \| Private \| Public \| \| --- \| --- \| --- \| \| Urbanization \| 2 \| -2 \| \| Rural revival \| -2 \| 2 \| \| Mass growth \| 0 \| 0 \|   Demographics 🡪 Rural economy   \|  \| Intensive agriculture \| Diversified & regenerative agriculture \| \| --- \| --- \| --- \| \| Mass growth \| 1 \| -1 \|   Approach to infra 🡪 Water quality   \|  \| Improved \| Poor \| \| --- \| --- \| --- \| \| Natural \| -1 \| 1 \|   Approach to infra 🡪 Ecological integrity   \|  \| High \| Low \| \| --- \| --- \| --- \| \| Distributed \| 1 \| -1 \|   Data systems 🡪 Approach to infra for resilience   \|  \| Centralized \| Distributed \| Natural \| \| --- \| --- \| --- \| --- \| \| Patchwork \| 3 \| -1 \| -2 \|   Government investment approach 🡪 Approach to infra for resilience   \|  \| Centralized \| Distributed \| Natural \| \| --- \| --- \| --- \| --- \| \| Enhanced \| -3 \| 1 \| 2 \| \| Reactive \| 3 \| -1 \| -2 \| |

**S5 – Robust scenarios**

The scenario tableau in Figure E1 depicts the eight scenarios that are robust to at least two versions (a, b, c) of both prototypes 1 AND 2, in addition to nine additional scenarios that are robust to all three versions (a, b, c) or prototype 1 OR 2.


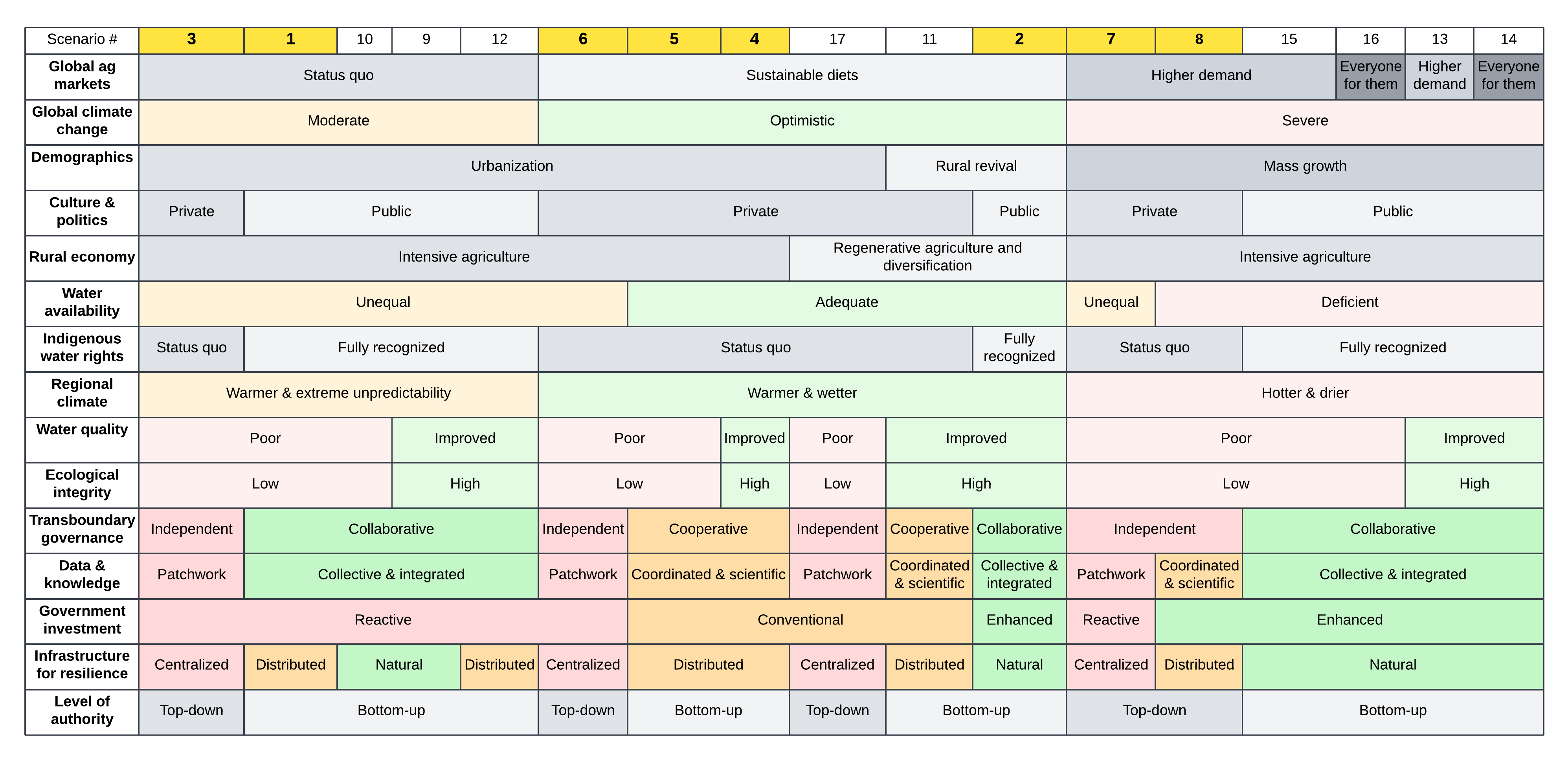


Figure E1: Scenario tableau depicting 17 robust scenarios according to looser robustness criteria. Original eight robust scenarios highlighted in yellow at the top of the tableau.

**S6 – WORKSHOP PROTOCOL**

**Presentation of study results**

**Breakout session 1**

*Explanation in full group:*

Thank you for your attention during that presentation! We are now ready to move on to our first breakout session.

The purpose of this breakout session is to explore your assumptions about the future of the Red River Basin through the process of ranking the scenarios I just showed you according to their: 1) desirability, and 2) plausibility.

In this breakout, you will split into breakout groups of 4 to 5 participants. Each group will be joined by a facilitator. You will have access to the 4 to 6 distinct scenarios, each with a scenario ‘narrative’ and a landscape sketch.

Your group will be given [X] minutes to:

1. **Rank X scenarios from most to least desirable**
2. **Rank X scenarios from most to least plausible**

Please keep in mind:

- Facilitator will be present to facilitate and will use a Miro board to assist, which is a virtual equivalent of a flipchart and sticky notes
- If you are not finished ranking based on desirability after ~15-20 minutes that is okay. Your facilitator will move your group to the plausibility ranking.
- Don’t simply state a ranking, discuss the reasons why you chose certain scenarios as more or less desirable/plausible than others in as much detail as possible. Your facilitator may ask follow up questions to find out this *why*.
- Like I said earlier on, this exercise is not about coming up with the “right” ranking (it may not exist!) but about exploring the future and our assumptions. The process may surface very different opinions or desires about the future, and may expose trade-offs between different goals. Try to be curious, respectful, and open to other perspectives.

Be prepared to choose one person to report back your reflections on the exercise. The report back will focus on interesting assumptions and themes that surfaced, not on your final ranking of the scenarios.

With that, I will send you off to breakout rooms.

*In breakout rooms:*

*Facilitator explains the exercise again and shares their screen with a Miro board. The Miro board will depict the 4-6 scenarios with brief descriptions. Facilitators and participants will have access to longer descriptions presented in the introduction if they need them.*

*Participants try to rank scenarios from most to least desirable and facilitator will use numerical indicators to follow discussion (or move scenarios around on the board). Facilitators will ask follow-up questions as needed to find out the rationale behind rankings. Participants may agree on the ranking, but it is perfectly fine if they do not (e.g., there could be two scenarios ranked ‘most desirable’ by different participants).*


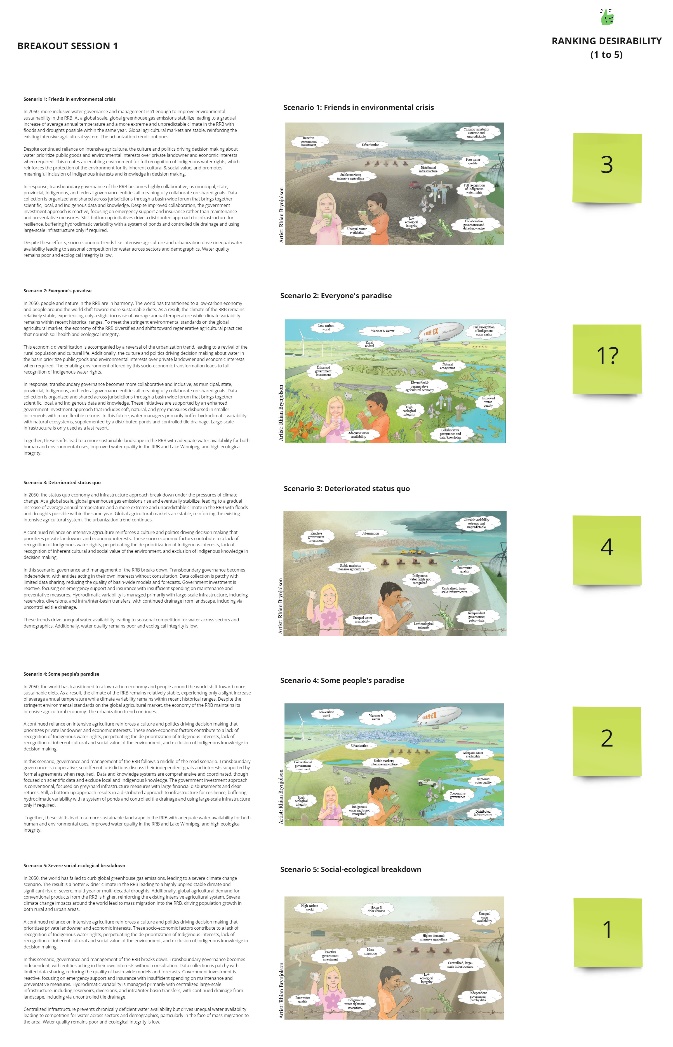

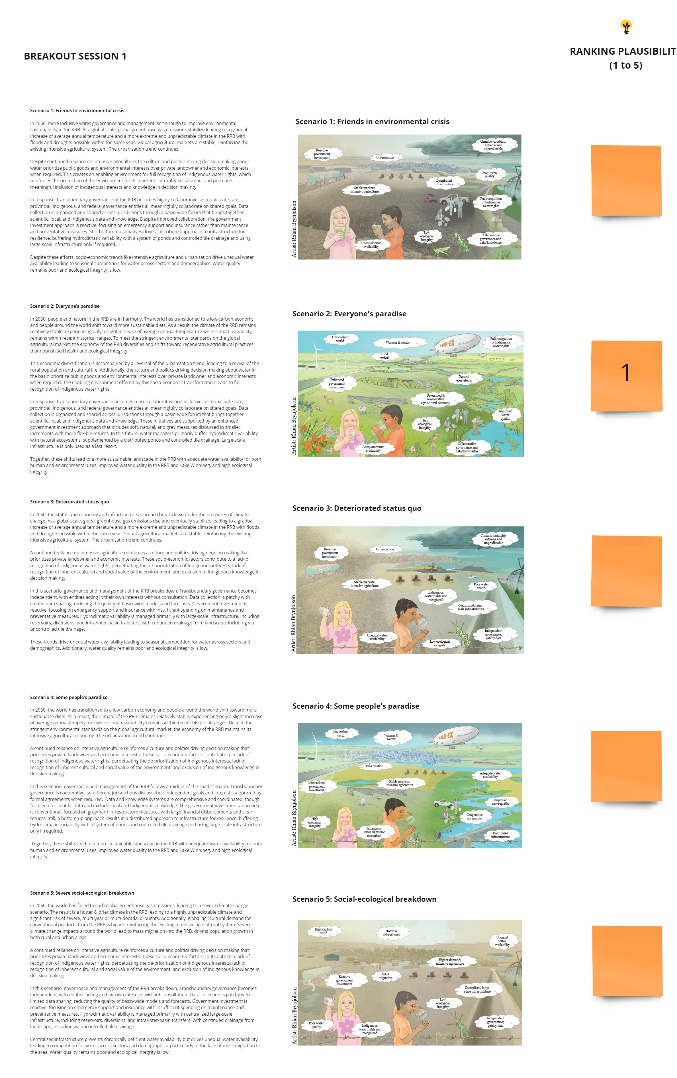


Figure 1: Miro board for Breakout session #1

*Report back in full group:*

*Breakout rooms are closed and participants return to the main Zoom room.*

I will now ask a representative from each group to give a 1 to 2 minute summary of the main themes and insights from your group. Don’t’ worry about the ranking itself, just broader insights. One of our facilitators is going to time you and stop you at 2 minutes.

Thank you so much for your engagement in this part of the session. We will now take a short [5-10] minute break, so see you back at X:XX.

---- BREAK ----

**Breakout session 2**

*Explanation in full group:*

We are now moving into our second breakout session. The purpose of this breakout is to connect these long term scenarios to actions we are taking to build resilience in the present.

During our interviews, I asked participants for examples of initiatives that are happening now that they think will contribute to a resilient future. I have gathered some together here.

During your breakout session, your facilitator will lead you through the following steps:

1. **Choose one example from the preloaded stickies on the Miro board.**
2. **Discuss as a group which scenarios that initiative is promoting, *and why*. In other words, which scenarios become MORE likely by pursuing this initiative?** For example, if I was interested in building a large piece of grey infrastructure, I might start looking for scenarios with ‘centralized infrastructure’ as the dominant approach to infrastructure for resilience, and see what kind of scenarios are associated with that.
3. **Discuss as a group which scenarios that initiative is inhibiting, *and why*. In other words, which scenarios become LESS likely by pursuing this initiative?**
4. **Choose another example from the list of initiatives and report step 2 (if time)**

*In breakout rooms:*

*Facilitator explains the exercise briefly again and shares their Miro board with pre-loaded sticky notes of ‘initiatives’. Participants decide on an initiative to start with. Facilitator then moves that sticky over to the Miro board of scenarios and leads participants through discussing which scenarios that initiative is promoting versus inhibiting, and why.*

*(if time) With 5 minutes remaining, the facilitator stops the exercises and asks the group to discuss:* ***What does this exercise tell us about our near-term efforts to build resilience in the RRB?***

***
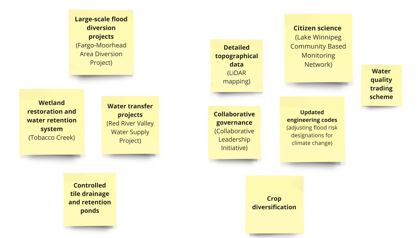
***

Figure 2: Miro board for breakout session #2

*(if time)* *Debrief in full group:*

Each group please take a minute to share which initiative you chose and any insights from you discussion about which scenarios it is promoting and inhibiting. One of our facilitators is going to time you and stop you at 2 minutes.

**Full group debrief**

We have two questions that will discuss for the remainder of our time together:

- Did this process of scenario development and discussion change the way you think about the future of the RRB? Why or why not? How?
- What are the implications of this discussion for the way we “build resilience” in the present?

**Closing**

**S7 – STRATEGY ASSESSMENT**

Table D1: Influence judgments changed for strategy assessment, differentiated from prototype 1 (a, b,c)

| Prototype | Version | Purpose | Rationale | Judgment sections |
| --- | --- | --- | --- | --- |
| 3 | a  b  c | To evaluate collaborative response to drought | The most direct influence that evaluates a collaborative response to drought is to adjust the influence of water availability on transboundary governance. In this strategy, deficient or unequal water availability promotes – rather than restricts – collaborative and cooperative governance. Additionally, the assumption that deficient or unequal water availability promotes top-down centralization of authority was removed to avoid contradicting these positive outcomes. | Water availability 🡪 Transboundary governance   \|  \| Collaborative \| Cooperative \| Independent \| \| --- \| --- \| --- \| --- \| \| Adequate \| -3 \| 1 \| 2 \| \| Unequal \| 1 \| 1 \| -2 \| \| Deficient \| 2 \| 1 \| -3 \|   Water availability 🡪 Level of authority   \|  \| Bottom-up \| Top-down \| \| --- \| --- \| --- \| \| Adequate \| 0 \| 0 \| \| Unequal \| 0 \| 0 \| \| Deficient \| 0 \| 0 \| |
| 4 | a  b  c | To evaluate implications of a true market for ecological goods and services | Under a true market for ecological goods and services, both private and public interests would promote a diversified and regenerative agricultural economy, because the additional ecological goods and services offered by regenerative agriculture would be valued in the economy. Also, the restricting influence of private interests on distributed and natural infrastructure approaches would flip to promoting influences, as landowners would benefit from the value of the ecological goods and services offered by these approaches. | Cultural & political drivers 🡪 Rural economy   \|  \| Intensive agriculture \| Diversified & regenerative agriculture \| \| --- \| --- \| --- \| \| Private \| -2 \| 2 \| \| Public \| -3 \| 3 \|   Cultural & political drivers 🡪 Approach to infra for resilience   \|  \| Centralized \| Distributed \| Natural \| \| --- \| --- \| --- \| --- \| \| Private \| -3 \| 1 \| 2 \| \| Public \| -2 \| -1 \| 3 \| |
| 5 | a  b  c | To evaluate the implications of effective demand management on water availability | Effective demand management reduces water demand, thereby reducing anthropogenic pressures on water availability. The two anthropogenic influences on water availability in the model are water use from the rural economy and demographics. The influence of intensive agriculture on water availability was zeroed to reflect no significant influence, whereas regenerative agriculture improves water availability as the soil quality improves its water storage capacity. The influence of water availability on the state of the rural economy was also zeroed, because demand management would no longer be a source of selectivity for the economy. | Rural economy 🡪 Water availability   \|  \| Adequate \| Unequal \| Deficient \| \| --- \| --- \| --- \| --- \| \| Intensive agriculture \| 0 \| 0 \| 0 \| \| Diversified & regenerative agriculture \| 3 \| -1 \| -2 \|   Water availability 🡪 Rural economy   \|  \| Intensive agriculture \| Diversified & regenerative agriculture \| \| --- \| --- \| --- \| \| Adequate \| 0 \| 0 \| \| Unequal \| 0 \| 0 \| \| Deficient \| 0 \| 0 \|   Demographics 🡪 Water availability   \|  \| Adequate \| Unequal \| Deficient \| \| --- \| --- \| --- \| --- \| \| Urbanization \| 0 \| 0 \| 0 \| \| Rural revival \| 0 \| 0 \| 0 \| \| Mass growth \| 0 \| 0 \| 0 \| |

**S8 – Scenario narratives**

**Scenario 1: Friends in environmental crisis**

In 2050, more inclusive water governance and management isn’t enough to improve resilience in the RRB. At a global scale, global greenhouse gas emissions stabilize and the RRB experiences a gradual increase of average annual temperature and a more extreme and unpredictable climate, with floods and droughts more likely within the same year. Global agricultural markets are stable, reinforcing the existing intensive agricultural system. The urbanization trend continues.

Despite continued reliance on intensive agriculture, the culture and politics driving decision making about water prioritize public goods and environmental interests over private landowner and economic interests when required. This creates an enabling environment for full recognition of Indigenous water rights, which reinforces the protection of the environment for its inherent cultural & social value, and promotes meaningful inclusion of Indigenous interests and knowledge in decision making.

In response, transboundary governance of the RRB becomes highly collaborative, as municipal, state, provincial, Indigenous, and federal governance entities all meaningfully collaborate on shared goals. Data collection is organized and shared across jurisdictions through a basin-wide forum that brings together scientific, local, and Indigenous data and knowledge. Despite improved collaboration, the government investment approach is reactive, focusing on emergency support and insurance rather than maintenance and preventative measures. Still, bottom-up initiatives drive a distributed approach to infrastructure for resilience, buffering hydroclimatic variability with a system of ponds and controlled tile drainage and using large-scale infrastructure only if required.

Despite these efforts, socio-economic trends like intensive agriculture and urbanization drive unequal water availability leading to seasonal competition for water across sectors and demographics. Water quality remains poor and ecological integrity is low.

**Scenario 2: Everyone’s paradise**

In 2050, people and nature in the RRB are in harmony. The world has transitioned to a low-carbon economy and people around the world shift toward more sustainable diets. As a result, the climate of the RRB remains relatively stable, experiencing only a slight increase of average annual temperature while climate variability remains within recent historical ranges. To meet the stringent environmental standards on the global agricultural market, the economy of the RRB diversifies and shifts toward regenerative agricultural practices that nourish soil health and ecological integrity.

This economic diversification is accompanied by a reversal of the urbanization trend, leading to a revival of the rural population and cultural life. Additionally, the culture and politics driving decision making about water in the basin prioritize public goods and environmental interests over private landowner and economic interests when required. The enabling environment offered by this socio-economic transformation leads to full recognition of Indigenous water rights.

In response, transboundary governance becomes more collaborative and inclusive, as municipal, state, provincial, Indigenous, and federal governance entities all meaningfully collaborate on shared goals. Data collection is organized and shared across jurisdictions through a basin-wide forum that brings together scientific, local, and Indigenous data and knowledge. These initiatives are supported by an enhanced government investment approach that includes soft, natural, and gray measures disbursed in smaller increments with more flexible returns. In this future, water managers primarily buffer hydroclimatic variability with natural ecosystems, supplemented by a distributed ponds and controlled tile drainage. Large-scale infrastructure is only used as a last resort.

Together, these shifts lead to a more sustainable landscape in the RRB with adequate water availability for both human and environmental uses, improved water quality in the RRB and Lake Winnipeg, and high ecological integrity.

**Scenario 3: Deteriorated status quo**

In 2050, the status quo economy and infrastructure approach break down under the pressures of climate change. At a global scale, global greenhouse gas emissions rise and eventually stabilize, leading to a gradual increase of average annual temperature and a more extreme and unpredictable climate in the RRB with floods and droughts more likely within the same year. Global agricultural markets are stable, reinforcing the existing intensive agricultural system. The urbanization trend continues.

A continued reliance on intensive agriculture reinforces a culture and politics driving decision making that prioritizes private landowner and economic interests. These socio-economic factors contribute to a lack of recognition of Indigenous water rights, perpetuating the de-prioritization of Indigenous interests, lack of recognition of inherent cultural and social value of the environment, and exclusion of Indigenous knowledge in decision making.

In this scenario, governance and management of the RRB breaks down. Transboundary governance becomes independent, with entities acting in their own interests without consultation. Data collection is patchy with limited data sharing, reducing the quality of basin-wide models and forecasts. Government investment is reactive, focusing on emergency support and insurance with insufficient spending on maintenance and preventative measures. Hydroclimatic variability is managed primarily with large-scale infrastructure, including reservoirs, diversions, and intra/inter-basin transfers, with continued drainage from landscape, including via uncontrolled tile drainage.

These trends drive unequal water availability leading to seasonal competition for water across sectors and demographics. Additionally, water quality remains poor and ecological integrity is low.

**Scenario 4: Some people’s paradise**

In 2050, the world has transitioned to a low-carbon economy and people around the world shift toward more sustainable diets. As a result, the climate of the RRB remains relatively stable, experiencing only a slight increase of average annual temperature while climate variability remains within recent historical ranges. Despite the stringent environmental standards on the global agricultural market, the RRB maintains its intensive agricultural economy. The urbanization trend continues.

A continued reliance on intensive agriculture reinforces a culture and politics driving decision making that prioritizes private landowner and economic interests. These socio-economic factors contribute to a lack of recognition of Indigenous water rights, perpetuating the de-prioritization of Indigenous interests, lack of recognition of inherent cultural and social value of the environment, and exclusion of Indigenous knowledge in decision making.

In this scenario, governance and management of the RRB follows a middle-of-the-road scenario. Transboundary governance is cooperative, so different jurisdictions discuss their independent goals and interests supported by formal agreements when required.  Data and knowledge systems are comprehensive and coordinated, though focused on scientific data and exclude local and Indigenous knowledge. The government investment approach is conventional, focused on gray/hard infrastructure measures with large financial disbursements and clear returns. Still, a bottom-up approach results in a distributed approach to infrastructure for resilience, buffering hydroclimatic variability with a system of ponds and controlled tile drainage and using large-scale infrastructure only if required.

Together, these shifts lead to a more sustainable landscape in the RRB with adequate water availability for both human and environmental uses, improved water quality in the RRB and Lake Winnipeg, and high ecological integrity.

**Scenario 7: Severe social-ecological breakdown**

In 2050, the world has failed to curb global greenhouse gas emissions, leading to a severe climate change scenario. The result is a hotter & drier climate in the RRB leading to a highly unpredictable climate and significant risk of severe, multi-year or multi-decadal droughts. Additionally, global agricultural demand for conventional products from the RRB is higher, reinforcing the existing intensive agricultural system. Severe climate change impacts around the world lead to mass migration into the RRB, driving population growth in both rural and urban areas.

A continued reliance on intensive agriculture reinforces a culture and politics driving decision making that prioritizes private landowner and economic interests. These socio-economic factors contribute to a lack of recognition of Indigenous water rights, perpetuating the de-prioritization of Indigenous interests, lack of recognition of inherent cultural and social value of the environment, and exclusion of Indigenous knowledge in decision making.

In this scenario, governance and management of the RRB breaks down. Transboundary governance becomes independent, with entities acting in their own interests without consultation. Data collection is patchy with limited data sharing, reducing the quality of basin-wide models and forecasts. Government investment is reactive, focusing on emergency support and insurance with insufficient spending on maintenance and preventative measures. Hydroclimatic variability is managed primarily with centralized large-scale infrastructure, including reservoirs, diversions, and intra/inter-basin transfers, with continued drainage from landscape, including via uncontrolled tile drainage.

Centralized infrastructure prevents chronically deficient water availability but drives unequal water availability leading to competition for water across sectors and demographics, particularly in the face of mass migration to the area. Water quality remains poor and ecological integrity is low.

**S9 – Quotes from debrief workshop**

Table I.1: Key quotes from debrief indicating participant perspectives on the value of the transdisciplinary scenario exercise

| *Theme* | *Quotes* |
| --- | --- |
| Making sense of complexity | “I don’t think it changed how I thought about the basin, but… the scenario approach is just so effective. It presents a range, and you sort of look at these different gradations along the continuum and I just think it’s an excellent, excellent way to consider during complex situations.”  “… I think I was reminded [of]… the importance of that sustainable development perspective... When thinking about resilience in the Red River Basin it’s hard for me to picture or envision resilience in the basin without an acknowledgement of Indigenous rights going forward. It’s such a big part of the fabric... So, it’s just so key to have the social, environmental, and economic aspects that are brought together… these are hard decisions...” |
| Surface different perspectives | “For a long time, many in the U.S. have said we need to include more social science... But the truth is our projects often aren’t funded to do that and when the budget is tight that’s the part that gets dropped off and we really need to do more than that. And I’m trying to get that included in some projects I’m working on and being part of this has influenced some of my thinking on that to try harder on that aspect…”  “[We have] done a lot of work on kind of how decision-making is informed by both kind of facts and evidence but also perspectives… Being in these breakout groups is a good reminder that we all have priorities, biases, and just different places from which we’re coming to.”  “I think… there was a lot of enthusiasm for the discussion. These are just short, kind of scratch the surface. I think we could have probably spent hours. I’ve done a few scenario sessions and certainly the understanding that starts to happen when you get the group together and just sharing, understanding, and seeing the other perspectives.”  “I don’t know necessarily that everybody shares that same vision on how we are to get there and as I said earlier, not only is it different from province to province it’s different from country to country.”  “[It is] reassuring to see that there are these many different variables and many different perspectives in the basin and that… maybe we have some actual authority and power here to change the way our future is shaped.” |
| Affirming the value of collaboration | “I looked at all these faces that all have the same passion. We may see different priorities to reach that end goal, but the passion is definitely there for the basin.”  “It was kind of a reminder that by bringing people together… around scenarios like this may actually change the way our future is shaped and how we prioritize. Recognition of Indigenous rights… seems to be kind of coming through fairly clearly as a priority for many…”  “It reassured me… that we, in the basin, both in the United States and North Dakota and Minnesota and South Dakota part of the basin, we can work together.” |
